# Nuclear receptors as therapeutic targets in metabolic and cardiovascular disorders

**DOI:** 10.1016/j.isci.2025.114042

**Published:** 2025-11-14

**Authors:** Feifei Li, Qiujing Chen, Yang Dai, Lin Lu

**Affiliations:** 1 Department of Cardiovascular Medicine, Rui Jin Hospital, Shanghai Jiao Tong University School of Medicine, Shanghai 200025, P.R. China; 2 Institute of Cardiovascular Diseases, Shanghai Jiao Tong University School of Medicine, Shanghai 200025, P.R. China; 3 National Research Center for Translational Medicine, Rui Jin Hospital, Shanghai Jiao Tong University School of Medicine, Shanghai 200025, P.R. China

**Keywords:** Health sciences, Therapeutics, Biological sciences, Biochemistry

## Abstract

Nuclear receptors (NRs) are ligand-dependent transcription factors that play essential roles in maintaining metabolic homeostasis and regulating disease progression through the control of metabolism, inflammation, and cellular differentiation. Due to their central position in these pathways, NRs have emerged as critical therapeutic targets in a range of metabolic and cardiovascular disorders, including diabetes, metabolic dysfunction-associated steatotic liver disease ([MASLD]; previously termed non-alcoholic fatty liver disease [NAFLD]), and atherosclerosis. This review summarizes current understanding of the molecular mechanisms by which NRs regulate metabolic function, with particular emphasis on their contributions to disease pathogenesis. We further outline recent advances from preclinical and clinical studies that explore NR-targeted therapeutic strategies. A deeper understanding of NR biology in metabolic and cardiovascular contexts holds promise for the development of more effective and selective interventions.

## Graphical abstract

**Figure:**
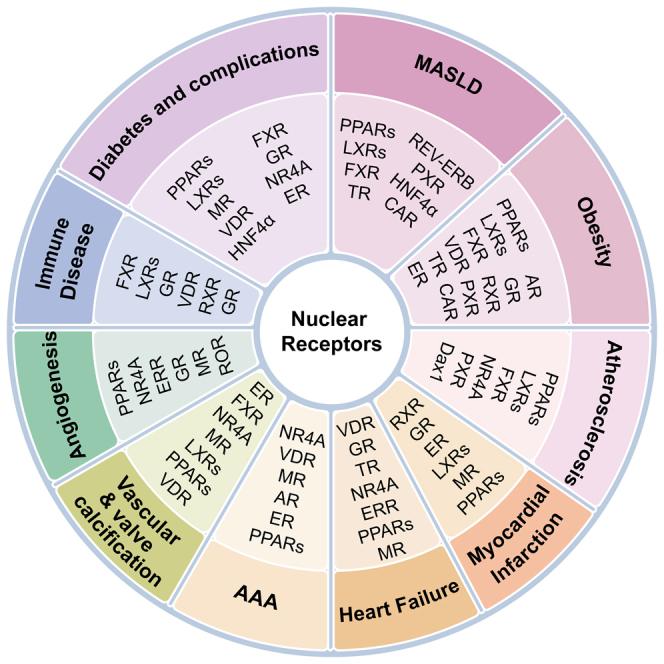

## Introduction

**Figure 1:**
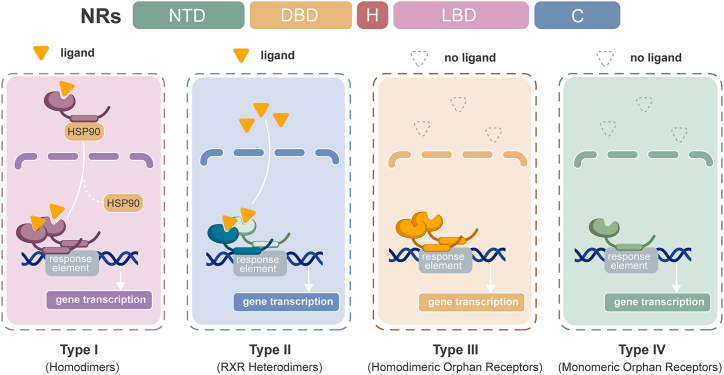
Classification and signaling mechanisms of NRs NRs share a conserved domain structure comprising the N-terminal (A/B) domain (NTD), DNA-binding domain (DBD), hinge region (H), ligand-binding domain (LBD), and C-terminal domain (C). They are classified into four mechanistic subtypes based on dimerization and ligand dependency. Type I receptors (such as GR and ER) form cytoplasmic complexes with HSP90 and translocate to the nucleus upon ligand binding. Type II receptors (such as PPARs and LXR) form heterodimers with RXR and activate transcription through ligand-induced cofactor exchange. Orphan receptors, lacking established endogenous ligands, function as homodimers (type III receptors) or monomers (type IV receptors), often through ligand-independent or constitutive activity.

Nuclear receptors (NRs) are a class of ligand-activated transcription factors that respond to various molecules, including hormones, lipids, and vitamins, to regulate diverse biological processes such as metabolism, immunity, and inflammation. The NR superfamily consists of 48 members in humans and is classified into seven subfamilies (NR0–NR6) based on sequence homology.^1^ Functionally, NRs are categorized by ligand identity into three groups: (1) endocrine receptors (such as estrogen receptor [ER], androgen receptor [AR], and glucocorticoid receptor [GR]), (2) adopted orphan receptors (such as peroxisome proliferator-activated receptors [PPARs], liver X receptors [LXRs], farnesoid X receptor [FXR], and pregnane X receptor [PXR]), and (3) orphan receptors (such as nuclear receptor subfamily 4 group A [NR4A] and DAX1) (Table 1).^2^^,^^3^^,^^4^^,^^5^ Mechanistically, NRs are also classified into four subtypes (types I–IV) based on dimerization and ligand dependency (Figure 1).^6^^,^^7^

**Table 1:** Human nuclear receptors

Nuclear receptors	Classification	Endogenous/synthetic ligands	Mechanism (genomic/non-genomic)	Effects	Disease associations
TRα (NR1A1)	Endocrine receptor	T3, T4^8^	Genomic: TR-RXR heterodimers at TREs^9^ Non-genomic: activation of NO/cGMP/PKG/ERK/Akt cascade^10^	Thermogenesis regulation^11^	Hypothyroidism^12^
TRβ (NR1A2)	Endocrine receptor	T3, T4,^8^ resmetirom,^13^ sobetirome,^14^ TG68^15^	Genomic: TR-RXR heterodimers at TREs^9^ Non-genomic: THRβ-PI3K/Akt/mTOR signaling^16^	Hepatic lipid/cholesterol regulation^11^	MASLD,^15^ MASH^13^
RARα/β/γ (NR1B1-3)	Endocrine receptors	Tretinoin, CD666, tamibarotene, trifarotene^8^	Genomic: RAR-RXR heterodimers at DR5, DR2, or DR1^8^	Energy balance, insulin resistance, cell differentiation,^17^ lipids metabolism, fibrogenesis^18^	Acute promyelocytic leukemia, diabetes, obesity,^17^ atopic dermatitis,^18^ fatty liver disease^19^
PPARα (NR1C1)	Adopted orphan receptor	Fatty acids, eicosanoids (LTB_4_, 8S-HETE, 20-HETE), phospholipids, polyphenols, fibrates (clofibrate, fenofibrate), GW9578, elafibranor^20^	Genomic: PPARα-RXR heterodimers at PPREs^20^ Non-genomic: activation of AMPK/Akt/eNOS/NO pathway^21^	Controlling inflammation and lipid metabolism pathways (FAO, gluconeogenesis)^20^	Atherosclerosis, diabetes, obesity, MASLD, MASH^20^
PPARβ/δ (NR1C2)	Adopted orphan receptors	Fatty acids, prostacyclin (PGI_2_), synthetic compounds (L-165,041, GW501516, Compound F)^22^	Genomic: PPARβ/δ–RXR heterodimers at PPREs^23^ Non-genomic: promotion of PI3K/Akt/eNOS signaling,^24^ inhibition of STAT3 via ERK1/2 and AMPK^25^	Regulation of lipid metabolism, cellular proliferation and the inflammatory response^22^	Primary biliary cholangitis,^26^ atherosclerosis,^27^ MASLD^28^
PPARγ (NR1C3)	Adopted orphan receptor	15 days, PGJ_2_, linoleic acid, oxLDL, thiazolidinediones (rosiglitazone, pioglitazone), GW9662, T0070907^22^	Genomic: PPARγ-RXR heterodimers at PPREs^29^ Non-genomic: inhibition of GPVI-stimulated platelet activation^30^	Regulation of obesity, insulin resistance, and inflammation^31^	T2D, MASLD/MASH,^32^^,^^33^ SLE, IBD^34^
REV-ERBα/β (NR1D1/2)	Orphan receptors	Heme, SR9009, SR9011^8^	Genomic: monomeric binding to RevRE or homodimeric binding to RevDR2^8^	Circadian rhythm, inflammation, fibrosis^35^	Atherosclerosis,^36^ abdominal aortic aneurysm,^37^ cardiac fibrosis,^38^ depression^39^
ROR α/β/γ (NR1F1-3)	Adopted orphan receptors	RORα/γ: cholesterol, SR1001, SR1078 RORα/β: CD2314 RORβ: tretinoin, all-trans-4-oxo-retinoic acid RORγ: XY101, SR2211^8^ RORα: SR3335 (ML-176)	Genomic: RORα: monomeric binding to ROREs (half-site) or homodimeric binding to Rev-DR2 sites RORβ: monomeric binding to ROREs RORγ: homodimeric binding to DR4 and DR5 elements^8^	Lipophagy, circadian rhythm regulation,^35^ autoimmunity (Th17 differentiation), cancer metabolism, glucose and lipid metabolism^40^	Autoimmune diseases, T2D,^40^ depression,^39^ atherosclerosis^41^
LXRα/β, (NR1H2/3)	Adopted orphan receptors	Oxysterols, T0901317, GW3965, SR9238, SR9243, TLC-2716,^42^ BMS-852927^43^	Genomic: LXRs-RXR heterodimers at LXREs^44^ Non-genomic: increasing NO production via LXRβ/Erα/eNOS signaling^45^	Controlling hepatic lipogenesis, reverse cholesterol transport,^46^ anti-inflammation^47^	Atherosclerosis,^48^ MASLD^49^
FXR (NR1H4)	Adopted orphan receptor	Bile acids, OCA, tropifexor, GW4064,^8^ cilofexor,^50^ nidufexor,^51^ HNC143, HNC180, and GW4064^52^	Genomic: FXR-RXR heterodimers at FXRE^53^ Non-genomic: activation of EGFR/ERK signaling,^54^ inhibition of cyclic nucleotide signaling in platelets^55^	Bile acid and cholesterol metabolism, absorption of dietary fats and vitamins^52^	MASH, primary biliary cirrhosis^52^
VDR (NR1I1)	Endocrine receptor	1,25-dihydroxyvitamin D3, calcitriol, alfacalcidol, paricalcitol^56^	Genomic: VDR-RXR heterodimers at VDRE^57^ Non-genomic: VDR-Src mediated Shh/Wnt/Notch signaling^58^	Bone and growth plate homeostasis,^59^ immune regulation,^60^ regulation of kidney function^61^	Osteoporosis, autoimmune disease,^56^ diabetic nephropathy^61^
PXR (NR1I2)	Adopted orphan receptor	Ginkgolide-A,^62^ rifampicin,^63^ SR12813^64^	Genomic: PXR-RXR heterodimer at PXRRE^65^ Non-genomic: negatively regulating platelet functions, thrombosis, and hemostasis via of Src-family kinases^64^	Detoxification, metabolism, inflammation inhibition, cell apoptosis, cell-cycle arrest, proliferation inhibition, tumor migration, and anti-oxidative stress^66^	Biliary cholestasis,^67^ MASLD, Crohn’s disease^62^
CAR (NR1I3)	Adopted orphan receptor	Diindoles,^68^ CITCO, TCPOBOP,^8^ DL5050,^69^ diazepam^70^	Genomic: CAR-RXR heterodimer at PBREM^65^	Drug metabolism, energy homeostasis, and cancer development^71^; bile acid homeostasis^72^; glucose and lipid homeostasis^73^	Cholestatic liver diseases,^74^ diabetes^73^
HNF4α (NR2A1)	Adopted orphan receptor	Linoleic acid, benfluorex, alverine^8^	Genomic: homodimeric binding to DR1^75^ Non-genomic: protein aggregates, ER expansion and autophagy^76^	FAO, lipophagy, lipid transport, bile acid uptake/synthesis,^35^ glycogen synthesis^77^	Diabetes, MASLD,^35^ renal Fanconi syndrome,^76^ ulcerative colitis, Crohn’s disease^78^
HNF4γ (NR2A2)	Adopted orphan receptor	Fatty acids^78^	Genomic: homodimeric binding to DR1^8^	Intestinal lipid malabsorption^79^	Obesity, T2D,^79^ IBD, ulcerative colitis^78^
RXRα/β/γ (NR2B1-3)	Adopted orphan receptors	Retinoic acid, fatty acids, bexarotene,^80^ CBt-PMN,^81^ LG100268^82^	Genomic: heterodimerization partner for other nuclear receptors^46^^,^^53^^,^^83^^,^^84^	Lipid, glucose, metabolism^80^	Atherosclerosis, obesity, hepatic fibrosis^80^
TR2/TR4 (NR2C1/2)	Orphan receptors	No confirmed endogenous ligand; TR4: nilotinib, genistein^85^	Genomic: TR2/TR4 homodimers or heterodimers binding to direct repeats of DNA elements^86^^,^^87^	Hematopoiesis,^88^ fertility, neuron development, metabolic regulation^89^	Metabolic syndrome, diabetes, atherosclerosis^89^
TLX (NR2E1)	Orphan receptors	Deorphanized by endogenous ligand; oleic acid,^90^ retinoids,^91^ tretinoin, BMS493^8^	Genomic: transcription regulation via TLX-binding sites with an AAGTCA sequence^92^	Neural development and adult neurogenesis, angiogenesis^93^	Neurological disorders and brain tumors^93^
PNR (NR2E3)	Orphan receptor	No confirmed endogenous ligand; biliverdin,^94^ photoregulin1^95^	Genomic: homodimeric binding to DR1^8^	Retinal development^96^	S-cone sensitivity syndrome, Goldmann-Favre syndrome, clumped pigmentary retinal degeneration, and retinitis pigmentosa^97^
COUP-TFI, COUP-TFII, EAR-2 (NR2F1/2/6)	Orphan receptors	No confirmed endogenous ligand	Genomic: COUP-TFs (homodimers or heterodimers with RXR) binding to repeat AGGTCA motifs^98^	Embryonic development, central nervous system development, cell proliferation, and tumorigenesis, adipogenesis, lipid homeostasis, and energy expenditure^99^^,^^100^	Bosch-Boonstra-Schaaf optic atrophy syndrome,^101^ congenital heart defects,^102^ congenital diaphragmatic hernia,^103^ MASLD^100^
ERα/β (NR3A1/2)	Endocrine receptors	Estriol, estrone, propylpyrazole triol, ethinylestradiol,^8^ SERMs (tamoxifen, raloxifene)^104^	Genomic: ERα/β homodimers at ERE,^105^ binding to DNA in conjunction with other transcription factors at composite elements; recruiting ERs to promoters via tethering^106^ Non-genomic: Src and downstream kinases in the Ras/Raf/MEK/ERK and PI3K/AKT/eNOS kinase cascades^107^	Vasodilation and insulin synthesis, cell proliferation and migration^107^^,^^108^	Breast cancer, atherosclerosis,^108^ Raynaud’s disease^103^^,^^104^^,^^109^^,^^110^
ERRα/β/γ (NR3B1-3)	Orphan receptors	No confirmed endogenous ligand Synthetic: XCT-790,^8^ SLU-PP-332, SLU-PP-915,^111^ 4-hydroxytamoxifen, DY131, GSK4716, GSK5182^112^	Genomic: ERRE-directed transcription (as a monomer or a homodimer or as a heterodimer with co-activators)^113^	Mitochondrial energy pathways,^114^ cardiac maturation,^115^ osteogenesis and vascular formation^116^	Heart failure,^115^ osteoarthritis,^112^ T2D^117^
GR(NR3C1)	Endocrine receptor	Cortisol, corticosterone, dexamethasone,^8^ CORT118335, CORT125385,^114^ CORT108297^118^	Genomic: direct GRE binding (GR homodimers or GR-MR heterodimers; monomeric half-site binding); and indirect binding (tethering)^119^^,^^120^ Non-genomic: enhanced ATP-induced Ca^2+^-mobilization, eNOS/NO pathway, activates Rho kinase, phosphorylation of Cav-1 and PKB/Akt in a Src-dependent manner^121^	Immunosuppression,^120^ hepatic glucose and lipid metabolism^114^	Inflammatory disorders, T2D,^120^ MAFLD/MASH^114^
MR(NR3C2)	Endocrine receptor	Deoxycorticosterone, aldosterone, cortisol, corticosterone, progesterone,^8^^,^^122^ spironolactone, eplerenone, finerenone^123^	Genomic: direct GRE/MRE binding (MR homodimers; MR-GR heterodimers; monomeric half-site—evidence limited)^122^^,^^124^ Non-genomic: increase cytosolic calcium, produce ROS, initiate inflammatory pathways via Rac1, NF-κB, and SGK1^125^	Inflammation and fibrosis^123^	Heart failure, hypertension,^123^ diabetic nephropathy^125^
PR (NR3C3)	Endocrine receptor	Progesterone, medroxyprogesterone, levonorgestrel, ORG2058, mifepristone, onapristone^8^	Genomic: homodimeric binding to PREs or monomeric half-site binding,^126^ binding to composite element,^127^ indirect binding via tethering^128^ Non-genomic: Src and downstream kinases in the Ras/Raf/MEK/MAPK and PI3K/AKT/mTOR/S6 kinase cascades,^107^ PI3K/Akt- and MAPK-eNOS pathways^129^	Vascular relaxation,^130^ proliferation, endocrine resistance, cancer stem cell expansion^131^	Atherosclerosis,^132^ breast, uterine, and ovarian cancers^131^
AR(NR3C4)	Endocrine receptor	Dihydrotestosterone, testosterone propionate, fluoxymesterone, mibolerone,^8^ GTx-024^133^	Genomic: direct ARE binding by AR homodimers,^134^ action at composite elements,^135^ indirect binding via tethering^136^ Non-genomic: Src and downstream kinases in the Ras/Raf/MEK/ERK and PI3K/AKT kinase cascades^107^^,^^137^	Cell proliferation and survival,^107^ endothelium functions,^137^ cardiac remodeling^138^	Prostate cancer,^136^ atherosclerosis,^139^ myocardial hypertrophy, heart failure^138^
Nur77, Nurr1, NOR1 (NR4A1-3)	Orphan receptors	No confirmed endogenous ligand Synthetic: Nur77: cytosporone B,^140^ BI1071,^141^ celastrol^142^ Nurr1: DHI,^143^ PGE1, PGA1,^144^ amodiaquine, chloroquine, cytosporone B^145^ NOR1: PGA2^146^	NR4A monomeric binding to NBRE and homo-/heterodimeric binding to NurRE; Nurr1/Nur77-RXR heterodimers at retinoic acid response elements,^84^ indirect binding via tethering^147^ Non-genomic: Nur77-mediated regulation of platelet activation via CAP1/AC/PKA pathway^148^	Proliferation, apoptosis, cellular stress, DNA repair endocrinology, neuronal signaling, hematopoietic, immune and metabolic processes^149^	Atherosclerosis,^150^^,^^151^ vascular calcification,^152^ heart failure,^153^ obesity, cancer^149^
SF-1 (NR5A1)	Adopted orphan receptor	Phospholipids (PIP_2_, PIP_3_),^154^ GSK8470,^155^ RJW100^156^	Genomic: transcriptional regulation via binding to consensus sequence^157^	Development and differentiation of steroidogenic tissues, sex determination, bile acids synthesis,^158^ energy homeostasis^159^	XY sex reversal and adrenal failure,^160^ obesity^161^
LRH-1(NR5A2)	Adopted orphan receptor	Phospholipids (DLPC, PIP_3_),^162^^,^^163^ GSK8470,^155^ RJW100^156^	Genomic: monomeric binding to consensus elements with/without FXR co-occupancy to regulate lipid metabolism genes,^164^ interacting with TCF4/β-catenin at target gene promoters (indirect DNA binding)^165^	Regulation of bile acid, cholesterol, and steroid hormone homeostasis^164^	T2D, MASLD/MASH,^114^^,^^166^ IBD^167^
GCNF (NR6A1)	Orphan receptor	No confirmed endogenous ligand	Genomic: higher-affinity homodimer binding (vs. monomer) at DR0 and at the extended half-site^168^	Germ cell development, embryonic development^169^	Oculovertebral renal syndrome^170^
DAX1 (NR0B1)	Orphan receptor	No confirmed endogenous ligand	Corepressor^171^^,^^172^	Autophagy, cholesterol metabolism, ion homoeostasis and transport, skeletal development^172^	Idiopathic central precocious puberty, adrenal insufficiency, lung adenocarcinoma, atherosclerosis^172^
SHP (NR0B2)	Orphan receptor	No confirmed endogenous ligand Synthetic: AHPN/3-Cl-AHPC^173^	Corepressor^174^	Autophagy/lipophagy^175^	MASH,^176^ obesity^177^

Structurally, NRs share a conserved domain structure comprising the N-terminal (A/B) domain, DNA-binding domain (DBD), hinge region (H), ligand-binding domain (LBD), and C-terminal domain (C)^6^^,^^7^ (Figure 1). The DBD typically contains two zinc finger motifs that recognize and bind to specific DNA sequences in the promoters or enhancers of target genes. The LBD is responsible not only for ligand recognition but also for direct interaction with co-regulatory proteins.^1^

Recent studies have revealed that NRs regulate metabolic homeostasis and disease progression through mechanisms beyond classical transcriptional regulation, including epigenetic modifications and protein-protein interaction networks. Aberrant expression or dysfunction of NRs has been strongly linked to a range of disorders, including diabetes, metabolic dysfunction-associated steatotic liver disease (MASLD), atherosclerosis, cancer, and inflammatory diseases, highlighting their importance as therapeutic targets.

**Figure 2:**
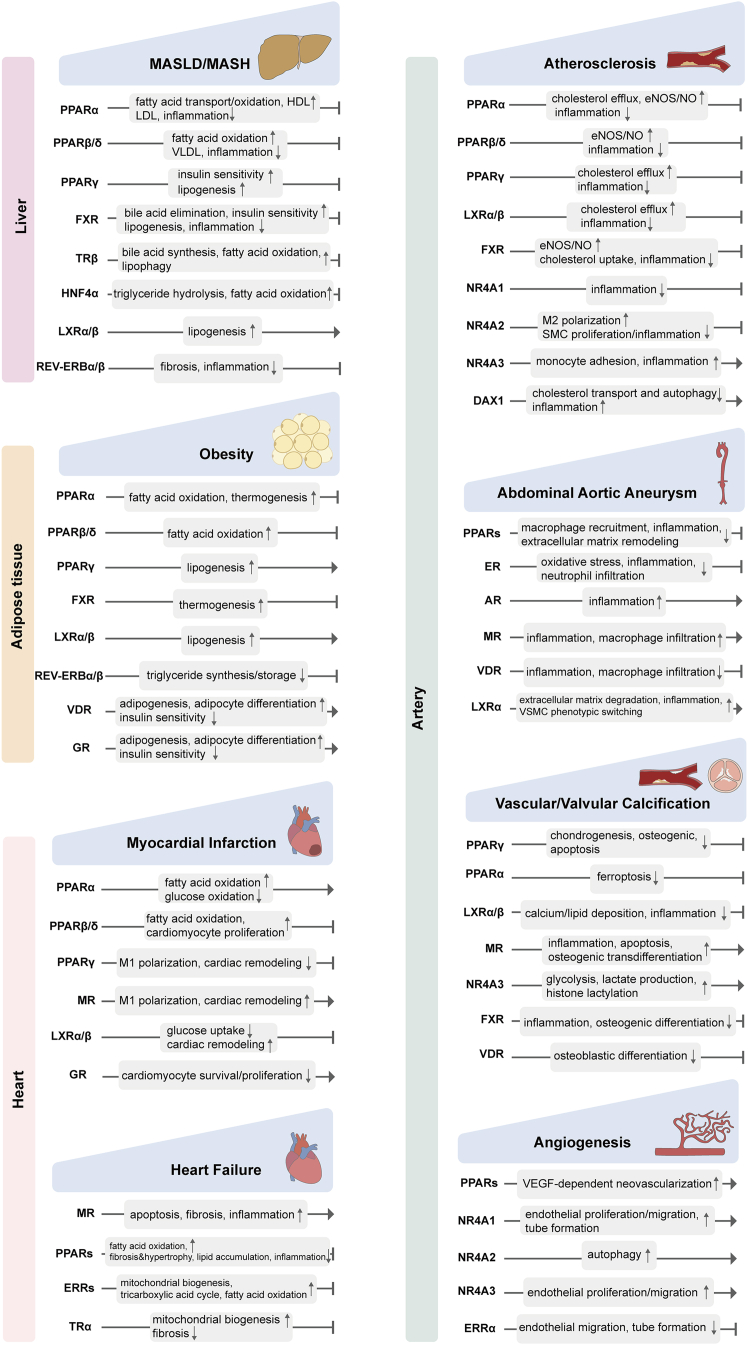
The roles of NRs in metabolic and cardiovascular disease NRs are expressed across multiple tissues, including the liver, adipose tissue, heart, and vasculature, where they direct tissue-specific programs of metabolism, inflammation, and remodeling. NR signaling is implicated in MASLD/MASH, obesity, MI, HF, atherosclerosis, AAA, vascular and valvular calcification, and angiogenesis.

In this review, we summarize the signaling pathways and metabolic functions of NRs, with a focus on recent advances in their roles in the pathogenesis of metabolic and cardiovascular diseases (Figure 2). We also provide an overview of emerging therapeutic strategies targeting NRs in both preclinical and clinical contexts (Table 2).

**Table 2:** Therapeutic strategies targeting NRs associated with metabolic and cardiovascular disorders

Nuclear receptors	Compounds/Drugs	Agonist/antagonist	Targeted disease	Preclinical/clinical
TRα/β (NR1A1/2)	Levothyroxine (LT4)/liothyronine (LT3)	Agonist	Hypothyroidism^178^	Clinical
TRβ (NR1A2)	Resmetirom (MGL-3196)	Agonist	MASLD/MASH^13^^,^^179^^,^^180^^,^^181^	Clinical
TRβ (NR1A2)	Sobetirome (GC-1)	Agonist	Dislipidemia,^14^^,^^182^ atherosclerosis,^183^ MASLD^184^	Clinical Preclinical Preclinical
TRβ (NR1A2)	TG68	Agonist	MASLD^185^	Preclinical
RARα/β/γ (NR1B1-3)	All-trans retinoic acid	Agonist	Acute promyelocytic leukemia,^186^ photoaging^187^	Clinical
RARβ/γ (NR1B2/3)	Tazarotene	Agonist	Psoriasis^188^	Clinical
RARγ (NR1B3)	Palovarotene	Agonist	Heterotopic ossification^189^	Clinical
RARα (NR1B1)	Tamibarotene	Agonist	Hepatitis B virus transcription^190^	Preclinical
RARγ (NR1B3)	Tectorigenin	Agonist	photoaged skin^191^	Preclinical
PPARα (NR1C1)	Fenofibrate	Agonist	Diabetic retinopathy,^192^ nonfamilial hypercholesterolemia,^193^ MASLD/MASH^194^^,^^195^	Clinical
PPARα (NR1C1)	Pemafibrate (SPPARMα)	Modulator	Dyslipidemia,^196^ T2D,^197^ MASLD^198^	Clinical
PPARβ/δ (NR1C2)	Seladelpar	Agonist	Primary biliary cholangitis^26^	Clinical
PPARγ (NR1C3)	Rosiglitazone	Agonist	T2D^199^	Clinical
PPARγ (NR1C3)	Pioglitazone	Agonist	T2D,^200^ MASLD/MASH,^32^^,^^33^	Clinical
PPARγ (NR1C3)	GW9662	Antagonist	MASLD/MASH^201^^,^^202^	Preclinical
PPARγ	INT131 (SPPARMγ)	Modulator	T2D^203^	Clinical
Dual PPARα/δ (NR1C1/2)	Elafibranor	Agonist	MASLD,^204^ primary biliary cholangitis^205^	Clinical
Dual PPARα/γ (NR1C1/3)	Saroglitazar	Agonist	MASLD/MASH^206^	Clinical
Pan-PPAR (NR1C1/2/3)	Lanifibranor	Agonist	MASLD/MASH^207^^,^^208^	Clinical
REV-ERBα/β (NR1D1/2)	SR9009/SR9011	Agonists	Circadian rhythm, obesity^209^ atherosclerosis,^36^ cardiac fibroblasts^38^	Preclinical
RORα	SR3335 (ML176)	Inverse Agonist	T2D^210^	Preclinical
RORα/γ (NR1F1/3)	SR1001	Inverse Agonist	Atherosclerosis^41^	Preclinical
LXRβ (NR1H2)	BMS-852927	Agonist	Hypercholesterolemia^43^	Clinical
LXRα/β (NR1H2/3)	T0901317	Agonist	T2D,^211^ atherosclerosis,^212^ MS^213^	Preclinical
LXRα/β (NR1H2/3)	GW3965	Agonist	T2D,^214^ atherosclerosis,^212^^,^^215^ myocardial ischemia/reperfusion injury^216^	Preclinical
LXRα/β (NR1H2/3)	DMHCA	Agonist	T2D,^217^ atherosclerosis^218^	Preclinical
LXRα/β (NR1H2/3)	SR9238	Inverse Agonist	MASLD/MASH^42^^,^^219^^,^^220^	Preclinical
LXRα/β (NR1H2/3)	SR9243	Inverse Agonist	MASLD/MASH^42^^,^^221^	Preclinical
LXRα/β (NR1H2/3)	TLC-2716	Inverse Agonist	Dyslipidemia^42^	Clinical
FXR (NR1H4)	Obeticholic acid	Agonists	Primary biliary cholangitis,^222^ MASH^223^	Clinical
FXR (NR1H4)	Tropifexor	Agonists	MASH,^224^ primary biliary cholangitis^225^	Clinical
FXR (NR1H4)	Cilofexor	Agonists	MASH,^50^ primary sclerosing cholangitis^226^	Clinical
FXR (NR1H4)	Nidufexor	Agonistic	MASH^227^	Clinical
FXR (NR1H4)	GW4064	Agonists	Atherosclerosis,^228^ MASH^229^	Preclinical
FXR (NR1H4)	EDP 305	Agonists	MASH^230^	Clinical
FXR (NR1H4)	TERN-101	Agonist	MASH^231^	Clinical
FXR (NR1H4)	EYP001	Agonist	MASH^232^	Clinical
FXR (NR1H4)	GUDCA	Antagonist	T2D^233^	Preclinical
FXR (NR1H4)	Glycine-β-MCA	Antagonist	Obesity^234^	Preclinical
FXR (NR1H4)	Theonellasterol	Antagonist	Obstructive cholestasis^235^	Preclinical
VDR (NR1I1)	Vitamin D	Agonist	Osteoporosis^236^	Clinical
VDR (NR1I1)	Alfacalcidol	Agonist	Osteoporosis,^237^ hypoparathyroidism^238^	Clinical
VDR (NR1I1)	Paricalcitol	Agonist	Diabetic dephropathy^61^^,^^239^	Clinical
PXR (NR1I2)	Rifampicin	Agonist	Pruritus in liver diseases^240^	Clinical
CAR (NR1I3)	TCPOBOP	Agonist	T2D^73^	Preclinical
HNF4α (NR2A1)	NCT	Agonist	Obesity, MAFLD/MASH^241^	Preclinical
RXRα/β/γ (NR2B1-3)	LG100268, LG101506	Agonists	T2D^82^	Preclinical
ERα/β (NR3A1/2)	Tamoxifen, raloxifene, lasofoxifene, arzoxifene	Antagonist	Osteoporosis^242^	Clinical
ERβ (NR3A2)	DPN	Agonist	Liver cirrhosis^243^	Preclinical
ERRα/β/γ (NR3B1-3)	SLU-PP-332, SLU-PP-915	Agonists	Heart failure^111^	Preclinical
ERRγ (NR3B3)	GSK5182	Inverse Agonist	Osteoarthritis,^112^ T2D^117^	Preclinical
GR (NR3C1)	Prednisone	Agonist	SLE,^244^ RA^245^	Clinical
GR (NR3C1)	Prednisolone	Agonist	RA^246^	Clinical
GR (NR3C1)	Methylprednisolone	Agonist	SLE,^247^ MS,^248^ IBD^249^	Clinical
GR (NR3C1)	Dexamethasone	Agonist	SLE,^250^ RA^251^	Clinical
GR (NR3C1)	Mifepristone	Antagonist	Cushing’s syndrome^252^	Clinical
GR (NR3C1)	Relacorilant	Antagonist	Cushing’s syndrome^253^	Clinical
GR (NR3C1)	CORT108297/CORT118335 (SGRMs)	Modulator	MASLD,^254^ obesity^255^	Preclinical
MR (NR3C2)	Spironolactone	Antagonist	CKD,^256^ heart failure,^257^ hypertension^258^	Clinical
MR (NR3C2)	Eplerenone	Antagonist	Heart failure,^259^ myocardial infarction,^260^^,^^261^ hypertension^262^	Clinical
MR (NR3C2)	Esaxerenone (CS-3150)	Antagonist	Hypertension,^262^ T2D, microalbuminuria^263^	Clinical
MR (NR3C2)	Finerenone	Antagonist	Heart failure,^264^^,^^265^ CKD with T2D^266^^,^^267^	Clinical
MR (NR3C2)	SM-368229	Antagonist	Hypertension^268^^,^^269^	Preclinical
MR (NR3C2)	PF-3882845	Antagonist	Hypertension^270^	Clinical
MR (NR3C2)	KBP-5074	Antagonist	Hypertension^271^	Clinical
Nur77 (NR4A1)	Csn-B	Agonist	Intestinal fibrosis,^272^ acute cardiac allograft rejection,^273^ multiple sclerosis^274^	Preclinical
LRH-1(NR5A2)	DLPC	Agonist	T2D, MASLD/MASH^166^	Preclinical

NCT, N-*trans*-caffeoyltyramine.

### Physiological functions of NRs

By sensing endogenous ligands such as lipids, bile acids, and steroid hormones, NRs regulate key pathways in glucose and lipid metabolism, including insulin sensitivity, lipid storage, and energy expenditure. In parallel, NRs modulate inflammatory responses by controlling the activation, polarization, and resolution of immune cells. Through their integrative functions, NRs serve as critical molecular links between metabolic and immune regulation.

#### Glucose metabolism

Numerous evidence suggests that several metabolically active NRs, including PPARs, LXRs, and FXR, regulate glucose metabolism by modulating key enzymes, glucose transporters, and hormonal signaling pathways. These NRs have emerged as important therapeutic targets for diabetes and its complications.

##### PPARs

The PPAR family consists of three homologous isoforms: PPARα (NR1C1), PPARβ/δ (NR1C2), and PPARγ (NR1C3). PPARα is ubiquitously expressed, with particularly high levels in the liver, heart, and adipose tissue, where it regulates fatty acid transport, esterification, and oxidation. PPARβ/δ is mainly expressed in the skeletal muscle as well as adipose tissue, with lower expression in the liver; it contributes to fatty acid oxidation (FAO) and glucose uptake. PPARγ is highly expressed in adipose tissue and is a key regulator of adipocyte differentiation, lipid storage, and insulin sensitivity.^275^ Mechanistically, PPARs form a heterodimer with retinoid X receptor (RXR) and bind to PPAR response elements (PPREs) to activate the transcription of metabolism-related genes.^29^

PPARα improves insulin signaling through multiple mechanisms. PPARα alleviates insulin resistance by promoting FAO to reduce ectopic lipid accumulation,^276^ enhancing skeletal muscle glucose uptake via adiponectin-AMPK signaling,^277^ and suppressing pro-inflammatory pathways such as nuclear factor (NF)-κB to mitigate inflammation-induced impairment of insulin signaling.^278^ Loss or knockdown of PPARα leads to fasting-induced hypoglycemia and reduced hepatic glucose levels, highlighting its essential role in maintaining glucose homeostasis.^279^ PPARα regulates the transcription of key gluconeogenic enzymes, including phosphoenolpyruvate carboxykinase (PEPCK) and glucose-6-phosphatase (G6Pase), and its deficiency impairs hepatic glucose output, resulting in persistent hypoglycemia.^280^ Additionally, PPARα facilitates adaptive responses to fasting by activating the ketogenic transcriptional program through upregulation of the rate-limiting enzyme 3-hydroxy-3-methylglutaryl-CoA synthase 2.^281^

PPARβ/δ serves as a key regulator of metabolic and inflammatory pathways in insulin resistance and type 2 diabetes (T2D). It modulates adipose tissue macrophage polarization, thereby influencing systemic insulin sensitivity.^282^ Myeloid-specific PPARβ/δ deficiency leads to adipocyte dysfunction, insulin resistance, and hepatic steatosis.^283^ Mechanistically, PPARβ/δ activation protects skeletal muscle from palmitate-induced insulin resistance by upregulating β-oxidation genes such as Cpt1 and Pdk4 and enhancing AMPK phosphorylation.^284^ In the pancreas, PPARβ/δ controls β-cell mass and insulin secretion; its deficiency leads to islet hyperplasia and hyperinsulinemia.^285^ In skeletal muscle, PPARβ/δ promotes glucose uptake and reduces circulating glucose and insulin levels,^286^ promotes FAO, and suppresses glycolysis to prevent hypoglycemia.^287^

PPARγ regulates a network of genes critical for glucose homeostasis, including upregulation of glucose transporter type 4 (GLUT4), insulin receptor substrates 1 and 2 (IRS-1 and IRS-2), and c-Cbl-associated protein (CAP). It also modulates the expression of multiple adipokines—such as adiponectin, resistin, leptin, and tumor necrosis factor-α (TNF-α)—thereby influencing insulin sensitivity. In addition, PPARγ alleviates insulin resistance by modulating inflammation; mice with macrophage-specific PPARγ deletion exhibit impaired alternative macrophage activation, leading to insulin resistance and glucose intolerance.^288^ By regulating genes involved in insulin sensitivity and glucose transport, PPARγ improves both insulin resistance and hepatic gluconeogenesis.^289^^,^^290^

##### LXRs

LXRs consisting of two isoforms, LXRα and LXRβ, are ligand-activated transcription factors belonging to the NR superfamily. Although LXRα and LXRβ share high sequence homology, they differ in tissue distribution. LXRα is highly expressed in metabolically active tissues such as the liver, intestine, adipose tissue, and macrophages, whereas LXRβ is ubiquitously expressed. LXRs form heterodimers with RXR and bind to LXR response elements (LXREs) to regulate gene expression.^44^ LXRs play important roles in glucose metabolism, particularly in the liver, adipose tissue, and pancreatic β-cells, where they regulate key gene expression to maintain glucose homeostasis and insulin sensitivity. LXRs act as nutrient and glucose metabolism sensors upstream of ChREBP by modulating the expression of glucokinase (GK), nuclear O-GlcNAc signaling, and ChREBP activity.^291^ They enhance insulin sensitivity by repressing the hepatic expression of gluconeogenic genes, such as PEPCK and G6Pase, while upregulating GLUT4 in adipose tissue. Moreover, LXRs regulate insulin secretion and biosynthesis by modulating both glucose and lipid metabolism in pancreatic β-cells.^292^ In the liver, LXR activation induces GK expression and downregulates PGC-1α as well as other genes involved in gluconeogenesis, thereby contributing to the maintenance of glucose homeostasis.^293^

##### Nuclear receptor subfamily 4 group A

The NR4A family of orphan NRs, including NR4A1 (Nur77), NR4A2 (Nurr1), and NR4A3 (NOR1), is highly expressed in the liver, adipose tissue, skeletal muscle, and pancreatic β-cells. These receptors function as metabolic sensors that respond to stressors such as fasting and high-fat diet (HFD), thereby regulating glucose metabolism and insulin sensitivity.

Nur77 has been identified as a transcriptional regulator of glucose utilization in skeletal muscle and gluconeogenesis in the liver.^294^^,^^295^ Adenoviral overexpression of Nur77 induces gluconeogenic genes, stimulates glucose production, and increases blood glucose levels. Conversely, expression of a dominant-negative Nur77 mutant antagonizes these effects.^294^ Nur77-deficient mice exhibit increased susceptibility to diet-induced obesity and insulin resistance.^296^ Recent evidence shows that, in response to glucose stimulation, NR4A1 regulates the expression of metabolic genes involved in glucose transport (Glut2), mitochondrial energy metabolism (Ndufa4, Sdhb, Idh3g), and insulin synthesis (Ins1, Ins2); its deletion in β-cells impairs insulin secretion and disrupts glucose homeostasis.^297^ Nurr1 expression in skeletal muscle enhances glucose uptake and glycogen storage, providing protection against hyperglycemia.^298^ Deletion of NR4A1 and NR4A3 in β-cells leads to mitochondrial dysfunction and reduced insulin secretion.^299^ In adipocytes, NR4A3 overexpression enhances, whereas small hairpin RNA -mediated knockdown impairs, insulin-stimulated glucose transport and GLUT4 translocation and also attenuates insulin-induced phosphorylation of IRS-1 and Akt.^300^ These findings underscore the essential role of NR4A in modulating insulin sensitivity and glucose uptake.

##### Glucocorticoid receptor

GR plays a critical role in maintaining glucose homeostasis, primarily through the hepatic glucocorticoid-GR (GC-GR) axis. Glucocorticoids regulate gluconeogenesis by inducing the transcription of key enzymes, including tyrosine aminotransferase (TAT), PEPCK, G6Pase, and the glucose-6-phosphate transporter SLC37A4.^301^ Animal studies have shown that GR signaling contributes to hyperglycemia and diabetes development. Tissue-specific GR antagonism in the liver or adipose tissue reduces blood glucose levels.^302^^,^^303^^,^^304^^,^^305^ Moreover, β-cell-specific overexpression of NR3C1 (encoding GR) impairs insulin secretion and leads to glucose intolerance.^306^

#### Lipid metabolism

##### PPARs

PPARs control hepatic lipid metabolism. PPARα is the predominant isoform in the liver and governs the transcription of genes involved in lipid and lipoprotein metabolism, including lipoprotein lipase (LPL), apolipoproteins (APOA1, APOA2, APOA5), phospholipid transfer protein (PLTP), and genes related to fatty acid transport and oxidation (FABP1, FABP3, ACS, ACO, CPT1, CPT2).^307^ Activated by fatty acids released from triglyceride lipolysis, PPARα promotes fatty acid uptake, lipogenesis, and β-oxidation.^308^

PPARγ reduces circulating levels of triglycerides and free fatty acids while increasing high-density lipoprotein (HDL) cholesterol.^29^^,^^309^ It promotes adipocyte differentiation and facilitates fatty acid uptake and storage in lipid droplets, thereby limiting ectopic lipid deposition. Adipose-specific deletion of PPARγ2 impairs metabolic flexibility, reduces lipid storage in adipose, and redirects lipids to skeletal muscle, leading to intramuscular triglyceride accumulation.^310^

Hepatic PPARδ promotes FAO and lipogenesis to supply energy substrates for skeletal muscle, enhances glucose uptake and storage, and suppresses gluconeogenesis.^308^ PPARα and PPARδ exert similar effects on plasma lipoprotein profiles by increasing HDL cholesterol, lowering low-density lipoprotein (LDL) cholesterol and triglycerides, and reducing circulating free fatty acids. PPARδ also decreases apoC-III and increases apoA-II expression^311^ and may protect against hepatic steatosis by downregulating very-low-density lipoprotein (VLDL) receptor expression.^312^

##### LXRs

LXRα and LXRβ contribute differentially to cholesterol metabolism. In primary human macrophages, knockdown of LXRα cannot be compensated by LXRβ activation, resulting in impaired cholesterol efflux.^313^ Consistently, deletion of LXRα, but not LXRβ, leads to significant cholesterol accumulation in peripheral tissues and the liver,^314^ establishing LXRα as the predominant isoform in cholesterol regulation.^315^

LXRs promote reverse cholesterol transport (RCT) by upregulating ATP-binding cassette transporters (ABC) A1 and ABCG1, facilitating cholesterol efflux to HDL.^48^^,^^316^ Additionally, LXRs upregulate cholesterol 7α-hydroxylase (CYP7A1), enhancing cholesterol conversion to bile acids,^44^ and induce inducible degrader of the LDL receptor (IDOL), which reduces LDL receptor (LDLR) expression and decreases LDL uptake.^317^ Liver-specific LXRα deletion impairs RCT, cholesterol catabolism, and biliary excretion,^318^ while LXRβ^−/−^ mice on a high-cholesterol diet develop hypercholesterolemia,^319^ underscoring the importance of both isoforms in systemic cholesterol homeostasis.

Beyond lipid regulation, LXR activation exerts anti-inflammatory effects. LXRs inhibit key inflammatory signaling pathways by suppressing transcription factors such as NF-κB and STAT1 and by modulating the alternative splicing of MyD88 mRNA, thereby reducing the expression of pro-inflammatory genes.^320^^,^^321^^,^^322^^,^^323^

However, LXRα also activates sterol regulatory element-binding protein-1c (SREBP-1c), which induces the expression of lipogenic genes, including fatty acid synthase (FASN), acetyl-CoA carboxylase (ACC), and stearoyl-CoA desaturase 1 (SCD1), promoting hepatic fatty acid synthesis and contributing to triglyceride accumulation and hepatic steatosis.^44^ LXRα/β knockout mice exhibit impaired hepatic lipogenesis and are resistant to diet-induced obesity.^324^ Taken together, LXR activation has dual effects: it mitigates atherosclerosis by promoting RCT and suppressing inflammation, but drives hypertriglyceridemia and steatosis by inducing SREBP-1c-dependent hepatic lipogenesis.

##### FXR

FXR is highly expressed in the liver, intestine, and adipose tissue. Upon forming a heterodimer with RXR, FXR binds to FXR response elements (FXREs) to regulate the transcription of genes involved in bile acid and lipid metabolism.^53^ FXR suppresses CYP7A1, the rate-limiting enzyme in bile acid synthesis, by inducing the expression of small heterodimer partner (SHP), fibroblast growth factor 19 (FGF19), and its murine ortholog FGF15.^325^^,^^326^^,^^327^^,^^328^ Concurrently, FXR promotes bile acid elimination by inducing the expression of the organic solute transporter α/β (OSTα/β), which facilitates basolateral bile acid efflux and renal excretion.^329^^,^^330^ By coordinating bile acid synthesis and export, FXR activation protects against hepatic bile acid accumulation and toxicity.

FXR activation also lowers plasma cholesterol, triglycerides, and free fatty acids.^331^ FXR knockout models exhibit hepatic steatosis, hyperlipidemia, bile acid overload, inflammation, and fibrosis,^332^ and these phenotypes can be ameliorated by pharmacological FXR activation.^333^^,^^334^ FXR modulates cholesterol levels through multiple mechanisms. It suppresses the expression of proprotein convertase subtilisin/kexin type 9 (PCSK9), a negative regulator of the LDLR, thereby enhancing LDLR activity and potentiating the lipid-lowering effects of statins.^335^ FXR also upregulates scavenger receptor (SR) class B type I (SR-BI), promoting HDL-derived cholesterol uptake,^336^ and downregulates CD36, reducing hepatic lipid accumulation.^337^ In addition, FXR induces ABCG5 and ABCG8 to facilitate biliary cholesterol excretion.^338^ Collectively, FXR reduces intestinal cholesterol absorption and *de novo* synthesis while enhancing cholesterol clearance, improving systemic cholesterol balance.

Beyond its role in cholesterol metabolism, FXR also suppresses hepatic lipogenesis. FXR activation downregulates SREBP-1c and its lipogenic targets, including FASN, SCD1, and ACC, thereby reducing fatty acid synthesis and triglyceride accumulation and protecting against hepatic steatosis.^332^

##### Thyroid hormone receptors

Thyroid hormone (TH) receptors (TRs) also play essential roles in lipid homeostasis. TRα is predominantly expressed in the heart, brain, lungs, and bone, whereas TRβ is the primary isoform in the liver.^339^ TRs bind to TH response elements (TREs) in target gene promoters as a heterodimer with RXR.^9^ Knockout studies of TR isoforms have revealed isoform-specific metabolic functions: TRα1 is critical for maintaining thermogenesis, while TRβ is required for cholesterol regulation. Hepatic activation of TRβ induces systemic lipid-lowering effects, increases bile acid synthesis, and enhances FAO.^340^ Mice with mutations in TRα or TRβ display altered thyroid function; however, only TRβ-mutant mice exhibit elevated serum free fatty acids and triglycerides, along with hepatic lipid accumulation. In contrast, TRα1-mutant mice show reduced liver mass due to impaired lipid storage.^341^ TH regulates CYP7A1, a key enzyme in bile acid synthesis. Notably, TRβ-deficient mice fail to induce CYP7A1 in response to triiodothyronine (T3) and exhibit no cholesterol-lowering effect, indicating that TRβ mediates the lipid-regulatory actions of TH in liver.^342^

#### Immune regulation

Beyond metabolic regulation, NRs play pivotal roles in maintaining immune homeostasis. They coordinate the initiation and resolution of inflammation and influence immune cell differentiation and function.

##### GR

GR is a well-characterized anti-inflammatory NR. Both endogenous glucocorticoids (such as cortisol) and exogenous glucocorticoid drugs exert potent immunosuppressive and anti-inflammatory effects via GR activation. GRα mediates these effects through multiple mechanisms. As a homodimer, GRα binds glucocorticoid response elements (GREs) to induce anti-inflammatory genes such as lipocortin-1, serum leukoprotease inhibitor, and interleukin (IL)-10, and represses genes like osteocalcin through negative GREs. However, the predominant anti-inflammatory mechanism involves protein-protein interactions with transcription factors, particularly NF-κB and AP-1, leading to the suppression of pro-inflammatory cytokines including TNF-α, granulocyte-macrophage colony-stimulating factor, and IL-1β.^343^ In mouse models, GR suppresses inflammation mediated by macrophages, dendritic cells, and epithelial cells. It also impairs cytotoxic immune responses by downregulating interferon-γ (IFNγ) production and inhibiting the development of Th1 cells, CD8^+^ T cells, and natural killer (NK) cells.^344^

##### PPARs

All three PPARs isoforms exhibit immunomodulatory activity, generally exerting anti-inflammatory effects.^345^^,^^346^ Among them, PPARγ is recognized as a potent anti-inflammatory regulator. Upon activation, it suppresses pro-inflammatory cytokine expression by interfering with transcription factors such as NF-κB, signal transducer and activator of transcription (STAT), and activator protein-1 (AP-1).^347^ PPARγ also promotes macrophage polarization toward the anti-inflammatory M2 phenotype^348^ and attenuates allergic inflammation by inhibiting epithelial expression of adhesion molecules (vascular cell adhesion molecule-1 [VCAM-1] and intercellular adhesion molecule-1 [ICAM-1]), mucus-associated gene MUC5AC, and various chemokines.^349^^,^^350^ Additionally, PPARγ reduces neutrophil infiltration and myeloperoxidase activity in response to lipopolysaccharide (LPS)^351^ and inhibits eosinophil activation and degranulation induced by IL-5.^352^

Accumulating evidence indicates that PPARα counteracts inflammation through multiple mechanisms. PPARα also exerts anti-inflammatory effects through multiple mechanisms. It directly interacts with NF-κB, AP-1, and STAT, suppressing downstream gene expression in a DNA-independent manner. Notably, co-activation of PPARα and GR enhances repression of TNF-induced IL-6 transcription.^20^ Hepatocyte- and macrophage-specific PPARα studies show that PPARα suppresses pro-inflammatory gene expression. In hepatocytes, PPARα activation induces Il1rn and suppresses Il1rap and Il6ra. In macrophages, PPARα reduces IL-15 and IL-18 levels.^353^ Similarly, PPARβ/δ modulates inflammation by inhibiting NF-κB DNA binding, resulting in decreased levels of TNF-α, IL-1β, and IL-6.^354^

##### LXRs

LXRs possess anti-inflammatory and immunomodulatory properties. Activation of LXRs by synthetic ligands suppresses the expression of inflammatory genes in macrophages, including inducible nitric oxide synthase (iNOS), cyclooxygenase-2 (COX-2), and cytokines such as IL-6 and IL-1β, particularly in response to LPS or bacterial stimuli.^355^

One proposed mechanism involves LXRα-mediated regulation of cholesterol homeostasis. Ito et al. identified ABCA1 as a key mediator of LXR’s anti-inflammatory effects. ABCA1 induction attenuates inflammatory signaling by reducing cholesterol content in detergent-resistant membrane domains.^323^ Cholesterol accumulation in macrophages enhances IL-1β, IL-6, and TNF-α production, which in turn impairs cholesterol efflux. Endogenous activation of LXRα facilitates PPARγ/LXRα/ABCA1-mediated cholesterol efflux, while concurrently suppressing pro-inflammatory cytokine expression and downstream signaling.^315^

LXRα also inhibits NF-κB signaling,^356^ NLRP3 inflammasome activation,^357^^,^^358^ and TLR4- and TLR9-dependent signaling pathways.^359^^,^^360^^,^^361^ Post-translational modification, particularly SUMOylation, enhances the anti-inflammatory activity of LXRα.^362^ In addition, LXRα overexpression promotes M2 macrophage polarization, reinforcing its immunoregulatory role.^363^

##### FXR

FXR is predominantly expressed in the liver, intestine, and adrenal glands and is also present in innate immune cells such as macrophages, liver-resident Kupffer cells, NK cells, and dendritic cells. FXR signaling exerts anti-inflammatory effects by suppressing pro-inflammatory cytokine production, inhibiting inflammasome activation, and inducing anti-inflammatory mediators.^364^ In mouse models, FXR activation promotes an anti-inflammatory phenotype in hepatic macrophages, thereby ameliorating steatosis, inflammation, and fibrosis.^365^^,^^366^ In immune cells, FXR fosters a tolerogenic environment in both the liver and gut and attenuates leukocyte infiltration in models of colitis, acute hepatitis, and liver fibrosis.^367^^,^^368^^,^^369^^,^^370^

FXR mediates immunoregulatory effects through both SHP-dependent and -independent mechanisms. SHP, an atypical NR, lacks a DNA-binding domain and functions as a corepressor. FXR directly regulates SHP expression and facilitates the recruitment of other corepressors at the promoter of FXR target genes.^368^^,^^371^ SHP physically interacts with the c-Jun subunit of AP1, thus preventing its binding to inflammatory genes.^368^ Additionally, SHP represses chemokine CCL2 expression by inhibiting NF-κB subunit p65 activation of CCL2 promoter activity.^372^ In parallel, FXR modulates inflammation in an SHP-independent manner via the nuclear corepressor NCoR1: FXR can be recruited to promoter regions of pro-inflammatory genes (such as iNOS and IL-1β) and stabilizes the NCoR1 complexes, thereby blocking NF-κB binding and downregulating expression of these genes. Another anti-inflammatory action of FXR involves negative regulation of NLRP3 inflammasome assembly.^371^

##### Other NRs

The NR4A family has emerged as a key regulator of inflammation in disease progression. Dysregulated NR4A has been reported in inflamed synovial tissue, colorectal tumors, atherosclerotic lesions, and multiple sclerosis (MS).^373^ NR4A receptors are rapidly induced by inflammatory stimuli such as oxidized LDL (oxLDL) and LPS, functioning as mediators of inflammatory signaling.^374^^,^^375^^,^^376^ Several downstream targets of NR4A have been identified, including the inducible kinase IKKi and inflammatory cytokines such as IL-8, IL-17, and IFN-γ.^376^^,^^377^^,^^378^ Our previous study showed that glycated ApoA-IV induces NR4A3 expression and that NR4A3 deficiency attenuates atherosclerosis progression.^151^ Recently, we found that DAX1 deficiency suppresses M1 macrophage markers (such as TNF-α and IL-6), increases M2 markers (such as IL-4 and IL-10), and reduces macrophage infiltration in atherosclerotic lesions.^172^

#### Context-dependent regulation and pathway crosstalk

NRs act in a context-dependent manner shaped by tissue, sex, age, and disease stage. For example, PPARα serves as a master regulator of hepatic lipid metabolism, particularly by controlling fatty acid uptake and oxidation, as well as by influencing ketogenesis, triglyceride turnover, lipid droplet biology, gluconeogenesis, and bile acid synthesis/secretion^279^; however, cardiomyocyte-restricted PPARα activation can shift substrate use toward increased FAO and reduced glucose utilization and provokes a diabetic-like, lipotoxic cardiomyopathy phenotype, underscoring tissue divergence.^379^ Similarly, LXR activation promotes hepatic lipid accumulation and hypertriglyceridemia,^44^ whereas in macrophages, LXR protects against atherogenesis by inducing cholesterol efflux and repressing inflammatory gene expression.^212^ These examples underscore that whether tissue-selective targeting of NRs can achieve metabolic benefits while avoiding lipotoxicity and other adverse effects in non-target tissues remains to be determined.

Sex steroid receptor signaling, particularly ER and AR, exhibits sexual dimorphism in vascular biology and contributes to differences in endothelial function, vascular remodeling, and susceptibility to cardiovascular diseases such as atherosclerosis and hypertension.^380^ Clinical studies testing hormone modulation have yielded mixed results. For example, oral estradiol therapy initiated within 6 years after menopause was associated with less progression of subclinical atherosclerosis, whereas initiation ≥10 years after menopause provided no vascular benefit, supporting the “timing hypothesis” of hormone replacement therapy.^381^ In men, lower circulating testosterone and dihydrotestosterone concentrations in older age have been linked to higher cardiovascular risk and mortality, yet randomized controlled trials of testosterone therapy have not consistently demonstrated benefit, with some reporting improvements in surrogate markers, while others finding no effect or even progression of coronary atheroma.^382^ These discrepancies highlight unresolved questions regarding the context-specific vascular effects of sex hormones and underscore the need for carefully designed trials that account for age, baseline cardiovascular risk, and treatment timing.

In addition, disease progression modifies NR outputs. For example, LXR activation by agonists such as T0901317 not only inhibits the development of atherosclerosis in ApoE^−/−^ mice but also induces regression of advanced lesions by enhancing macrophage cholesterol metabolism and promoting CCR7-dependent egress of monocyte-derived macrophages from aortic plaques.^383^^,^^384^ Moreover, the “timing hypothesis” of hormone replacement therapy suggests that initiating oral estradiol therapy in early menopause slowed progression of carotid-artery intima-media thickness, whereas initiating therapy ≥10 years after menopause shows no such benefit,^381^ highlighting the importance of time since menopause and disease stage.

Crosstalk among major pathways further refines NR actions. PPARγ activation induces LXRα, which subsequently upregulates ABCA1 to promote cholesterol efflux, constituting the canonical PPARγ-LXR-ABCA1 axis.^385^ In bile acid metabolism, simultaneous activation of FXR and LXRα results in suppression of CYP7A1 mRNA, as the inhibitory effect of FXR overrides the stimulatory action of LXRα.^386^ At the level of protein-protein interactions, RXR functions as a central heterodimer partner for multiple NRs—including TRs, PPARs, LXRs, FXR, PXR, CAR, retinoic acid receptors (RARs), vitamin D receptor (VDR), Nurr1, and Nur77—to regulate target gene transcription.^46^^,^^53^^,^^83^^,^^84^ However, it remains unclear how different NRs compete or cooperate for shared partners such as RXR *in vivo* and whether these dynamic interactions can be selectively targeted without perturbing physiological homeostasis. Extending these insights, our recent work showed that Dax1 promotes atherosclerosis by interacting with LXR and the transcription factor EB (TFEB) to inhibit cholesterol transport and autophagy.^172^

NRs exert context-dependent effects also through crosstalk with non-NR signaling pathways. PPARγ ligands repress NF-κB-driven inflammatory gene expression in macrophages via a SUMOylation-dependent transrepression mechanism.^387^ Similarly, LXR activation not only promotes cholesterol efflux but also inhibits pro-inflammatory gene induction.^388^^,^^389^ In the gut-liver axis, FXR induces FGF15/19, which modulates CYP7A1 and links bile acid signaling.^327^

Taken together, these findings underscore that NR effects are highly context specific, contingent on the surrounding signaling milieu, tissue identity, and interactions with parallel receptor networks.

### NRs and disease

Given the central role of NRs in coordinating metabolic and immune pathways, dysregulated NR signaling is increasingly recognized as a major contributor to the pathogenesis of metabolic disorders. We outline the roles of specific NRs in the development of metabolic diseases such as diabetes, MASLD, and atherosclerosis, with a focus on their underlying mechanisms and therapeutic potential (Figure 2).

#### NRs in diabetes mellitus and its complications

Diabetes mellitus is a chronic metabolic disorder characterized by persistent hyperglycemia and commonly accompanied by complications such as nephropathy, retinopathy, and neuropathy. NRs play central roles in regulating glucose and lipid metabolism, inflammatory responses, oxidative stress, and cellular growth and differentiation. Consequently, dysfunction of NRs contributes to the pathogenesis of diabetes and its related complications.

##### PPARs

PPARs are involved in glucose and lipid metabolism as well as inflammation regulation and have been extensively studied in diabetes and its complications. PPARα activation improves renal morphology and function in ischemia-reperfusion injury models,^390^ suppresses inflammation in activated mesangial cells,^391^ and protects against diet-induced renal lipotoxicity.^392^^,^^393^ Increasing evidence supports a protective role for PPARα in diabetic nephropathy. PPARα knockout diabetic mice show accelerated disease progression, with mesangial expansion and increased albuminuria.^394^ The renoprotective effects of PPARα activation have been further confirmed in several experimental.^395^^,^^396^^,^^397^ PPARα expression is reduced in the diabetic retina,^398^^,^^399^ contributing to retinal inflammation and pathological neovascularization.^400^^,^^401^ Diabetic PPARα^−/−^ mice exhibit increased acellular capillaries formation, pericyte loss, and aggravated retinal neurodegeneration.^400^^,^^402^ Fenofibrate, a PPARα agonist commonly used to lower triglyceride, was shown to slow the progression of diabetic retinopathy in the Fenofibrate Intervention and Event Lowering in Diabetes (FIELD) study.^403^

PPARγ also confers renoprotective effects by suppressing the development and progression of diabetic nephropathy.^404^^,^^405^^,^^406^^,^^407^ In mesangial cells and proximal tubular epithelial cells, PPARγ agonists exhibit anti-proliferative, anti-fibrotic, and anti-inflammatory activities.^408^ In T2D models, PPARγ activation prevents glomerular endothelial dysfunction and podocyte injury, inhibits NF-κB activation, reduces reactive oxygen species (ROS) accumulation, and limits macrophage infiltration in renal tissue.^408^^,^^409^ PPARγ also upregulates LXRα and promotes cholesterol efflux in macrophages, contributing to renal lipid homeostasis and nephroprotection.^410^ Dual PPARα/γ agonists protects db/db mice from glomerular and tubulointerstitial injury and improves insulin sensitivity, glycemic control, and lipid profiles.^411^ Furthermore, genetic studies have linked the PPARγ Pro12Ala polymorphism to susceptibility to diabetic nephropathy.^412^^,^^413^

In diabetic retinopathy, PPARγ expression is suppressed under hyperglycemia, whereas activation by rosiglitazone delays disease progression,^414^ supporting its potential as a therapeutic target. Both PPARα and PPARγ have been proposed as candidates for treating diabetic retinopathy.^414^^,^^415^ Saroglitazar, a dual PPARα/γ agonist, not only improves hypertriglyceridemia, insulin sensitivity, and β-cell function^416^ but also appears to prevent retinopathy progression in diabetic patients.^417^

Compared to PPARα and PPARγ, PPARβ/δ has been less extensively studied in diabetic complications. PPARβ/δ is expressed in glomerular mesangial cells, the proximal tubules of the renal cortex and medulla, and stromal cells, where it promotes FAO and exerts anti-inflammatory and antioxidant effects,^407^ suggesting a potential renoprotective role. In type 1 diabetes models, including Akita and OVE26 mice, renal expression of PPARβ/δ is markedly reduced,^418^ and similar downregulation is observed in the proximal tubular cells of obese and diabetic rats exposed to oxidized lipids.^419^

##### LXRs

In diabetic animal models, LXR activation lowers blood glucose levels, enhances insulin sensitivity, inhibits hepatic gluconeogenesis, and reduces glucose output.^211^^,^^214^ Renal LXR expression is downregulated in type 1 diabetes models compared to controls.^418^ Activation of LXRα attenuates renal renin-angiotensin system activity in diabetic mice.^420^ As intracellular sterol sensors that regulate genes involved in cholesterol absorption, catabolism, and efflux, LXRs—particularly LXRα—promote cholesterol efflux via ABCA1 in glomerular mesangial cells, suggesting therapeutic potential in lipid-laden glomerular diseases.^410^^,^^421^ LXRβ deletion impairs urinary concentrating ability in mice, accompanied by reduced renal aquaporin-1 expression, decreased vasopressin-positive neurons in the hypothalamus, and downregulated urinary arginine vasopressin excretion.^422^ These findings underscore the role of LXRα in renal lipid metabolism and LXRβ in maintaining water and electrolyte homeostasis.

Inflammation and oxidative stress are key contributors to diabetic retinopathy. Elevated inflammatory cytokines have been observed systemically and in the retina.^423^^,^^424^ In streptozotocin (STZ)-induced diabetic models, LXR agonists inhibit retinopathy progression by downregulating oxidative stress-related genes and reducing retinal immune cell infiltration.^425^ The selective LXR agonist DMHCA restores retinal cholesterol homeostasis and improves retinal function in T2D.^217^

In diabetic neuropathy, hyperglycemia leads to abnormal lipid composition in peripheral nerve myelin, characterized by altered levels of phospholipids, cholesterol, and fatty acids, which compromise myelin integrity. LXR agonists improve nerve conduction velocity and nociceptive thresholds in diabetic rats by restoring SREBF-1c activity and upregulating myelin protein zero (P0), a key myelin structural component.^426^ Additionally, LXR activation reduces prostaglandin D2 production in dorsal root ganglia and alleviates neuroinflammation by modulating phosphatidylcholine and cholesteryl ester metabolism, thereby slowing neuropathy progression.^427^

##### Mineralocorticoid receptor

Activation of the mineralocorticoid receptor (MR) pathway plays a critical role in diabetic complications. MR enhances inflammasome assembly and vascular injury in T2D models.^428^ Myeloid-specific MR deletion improves glucose intolerance, insulin resistance, and hepatic steatosis via hepatocyte growth factor/Met signaling.^429^ MR contributes to diabetic nephropathy by promoting epithelial-mesenchymal transition and renal fibrosis, through mechanisms involving insulin resistance, microvascular dysfunction, mitochondrial impairment, oxidative stress, inflammation, and lipid abnormalities.^430^ MR antagonists (MRAs) have shown clinical benefits in reducing proteinuria in patients with diabetic nephropathy,^431^ with consistent findings from preclinical studies.^432^

In the retina, excessive MR activation promotes inflammation, oxidative stress, and vascular injury.^433^^,^^434^^,^^435^ MR is overexpressed in the retinas of both diabetic model and patients. Local MR antagonism attenuated key pathological features of diabetic retinopathy in Goto-Kakizaki (GK) rats, including retinal inflammation, vascular leakage, and macular edema at both early and advanced disease stages.^436^

##### VDR

VDR has demonstrated renoprotective effects in diabetic nephropathy. VDR expression is reduced in the kidneys of diabetic mice but increases following treatment with vitamin D analogs.^437^ In STZ-induced diabetes models, VDR-knockout mice exacerbate renal injury compared to controls.^438^ In diabetic retinopathy, studies have focused on VDR gene polymorphisms and disease susceptibility. In Korean patients with T2D, the B allele of the BsmI polymorphism (rs1544410) was associated with a lower risk of diabetic retinopathy.^439^ In contrast, Assis et al. reported no direct association but found that the B allele may influence glycemic control and insulin regulation in patients with diabetic retinopathy.^440^ In a Chinese cohort, the F allele of the VDR FokI polymorphism was linked to decreased diabetic retinopathy risk.^441^ A cross-sectional study further suggested that altered VDR polymorphism expression may contribute to microvascular complications in diabetes.^442^

##### Other NRs in diabetes

Hepatocyte nuclear factor 4 alpha (HNF4α) mutations are associated with maturity-onset diabetes of the young.^443^ Transient receptor potential canonical channel 1 (TRPC1), a target gene of HNF4α involved in mesangial cell contraction and glomerular function, is downregulated in patients with diabetic nephropathy, along with HNF4A expression, implicating this pathway in diabetic nephropathy pathogenesis.^444^

FXR activation exerts renoprotective effects in T2D mouse models, improving proteinuria, glomerulosclerosis, tubulointerstitial fibrosis, and macrophage infiltration.^445^^,^^446^ Conversely, Nr1h4 (the gene encoding FXR) deletion worsens renal injury in STZ-induced diabetic mice,^437^ suggesting FXR as a promising therapeutic target in diabetic nephropathy.

GR signaling is also involved in diabetic kidney injury. Podocyte GR is essential for maintaining glomerular endothelial cell homeostasis under diabetic condition.^447^ Endothelial-to-mesenchymal transition (EndMT), a key source of fibroblasts, contributes to renal fibrosis and progression to end-stage renal disease. Endothelial GR deficiency accelerates diabetic renal fibrosis via aberrant cytokine/chemokine expression, enhanced Wnt-dependent EndMT, and impaired FAO.^448^

Other NRs, including the NR4A subfamily (NOR1, NURR1, and Nur77), are markedly upregulated in the kidneys of diabetic C57BL/6 and db/db mice under hyperglycemic conditions.^449^ In addition, ER signaling has been implicated in diabetic nephropathy pathogenesis. ER pathway dysregulation is observed in diabetic kidneys,^450^ and ER activation mitigates diabetic nephropathy progression.^451^

#### NRs in MASLD

NRs are central regulators of hepatic metabolism, controlling genes involved in FAO, bile acid synthesis, and lipid transport, thereby coordinating hepatocellular lipid utilization.^452^

##### PPARs

PPARα expression is inversely correlated with the severity of metabolic dysfunction-associated steatohepatitis ([MASH]; formerly non-alcoholic steatohepatitis [NASH]), visceral obesity, and insulin resistance in humans.^453^ Hepatocyte-specific PPARα deletion induces hepatic steatosis, steatohepatitis, and hypercholesterolemia in mice.^454^ PPARα regulates genes involved in fatty acid transport, APO production, and β-oxidation^455^^,^^456^ and may suppress hepatic lipogenesis indirectly through coordination with LXR signaling.^44^^,^^456^ These effects contribute to increased HDL, decreased VLDL and LDL, and reduced hepatic triglyceride accumulation.^20^ Emerging evidence highlights the importance of the gut-liver crosstalk in modulating the progression of MASLD.^309^^,^^457^^,^^458^ Recent studies have identified an intestinal PPARα-fatty acid-binding protein 1 (FABP1) axis that regulates dietary fatty acid uptake and influences obesity and MASLD progression.^459^

PPARβ/δ is downregulated in the livers of patients with MASLD.^460^ Hepatic PPARβ/δ activation suppresses SREBP-1c,^461^ regulates the lipoprotein-related genes (VLDLR, ApoA5, ApoA4, and ApoC1), and enhances FAO, thereby reducing triglyceride and hepatic steatosis.^462^ PPARβ/δ-deficient mice fed an HFD exhibit hypertriglyceridemia due to excessive VLDL production and impaired LPL activity—both contributing to MASLD.^462^ Beyond lipid metabolism, PPARβ/δ modulates hepatic inflammation by promoting anti-inflammatory M2 polarization of Kupffer cells.^463^ Notably, the selective PPARβ/δ agonist seladelpar has shown efficacy in reducing hepatic inflammation and enhancing insulin sensitivity in MASH model.^464^

PPARγ promotes hepatic lipid accumulation by upregulating genes involved in lipid uptake, storage, and droplet formation, including FABP4, SREBP-1c, FSP27/Cidec, CD36, MGAT1, and PLIN2.^465^ Hepatocyte-specific deletion of PPARγ reduces hepatic steatosis in obese mice.^466^^,^^467^ Paradoxically, pharmacological activation of PPARγ improves features of MASLD, including hepatic steatosis, ballooning, and inflammation, and also the stage of fibrosis in non-diabetic, prediabetic, and T2D patients with MASLD.^468^ These benefits are attributed to enhanced lipid storage and redistribution and adipocyte differentiation, leading to reduced circulating levels of free fatty acids.^469^ PPARγ activation also promotes RCT and improves dyslipidemia^470^ and exerts anti-inflammatory and anti-fibrotic effects in non-parenchymal cells.^471^ These findings suggest that the tissue-specific actions of PPARγ may help explain how systemic PPARγ activation yields hepatoprotective effects and improves MASLD/MASH outcomes.

##### LXRs

LXRs exert a dual role in hepatic metabolism. On the one hand, LXRs promote RCT, facilitating cholesterol elimination via bile and feces.^472^ On the other hand, LXR activation directly upregulates SREBP-1c, FASN, SCD1, and ACC, which contributes to hepatic lipid accumulation and hypertriglyceridemia.^473^ In humans, hepatic LXR expression increases with MASLD severity.^474^ To address these challenges, Griffett et al. developed SR9238, a liver-specific LXR inverse agonist that significantly reduces hepatic steatosis, inflammation, and fibrosis in MASH models. SR9238 suppresses the expression of FASN, SREBP-1c, and SCD1, thereby lowering hepatic lipid accumulation.^219^ Similarly, Huang et al. observed that treatment with SR9243 attenuates hepatic inflammation and fibrosis in MASH, while also lowering circulating liver enzymes and LDL-C levels.^221^ In addition, several natural compounds—such as alpinetin, morin, luteolin, and curcumin—have been shown to inhibit LXRα transcriptional activity and downregulate SREBP-1c, exhibiting anti-steatotic effects in MASLD models.^475^^,^^476^^,^^477^^,^^478^

##### FXR

Hepatic FXR expression is reduced in patients with MASLD.^479^ Studies in tissue-specific knockout mice have shown that hepatic FXR regulates lipogenic genes and lowers hepatic triglyceride levels, thereby protecting against the onset of MASLD.^480^ Activation of FXR by obeticholic acid (OCA) alleviates hepatic steatosis and obesity in patients and experimental models of MASLD.^333^^,^^481^ Similarly, treatment with the FXR agonist GW4064 reduces hepatic steatosis and weight gain by suppressing the expression of CD36, a key lipid transporter.^337^ FXR activation improves metabolic outcomes through multiple mechanisms, including reductions in hepatic and plasma triacylglycerol levels, attenuation of inflammation, enhancement of insulin sensitivity, and protection against hepatotoxic insults.^482^ These lipid-lowering effects are mediated, at least in part, by inhibition of *de novo* lipogenesis via the FXR-SHP-SREBP-1c signaling axis.^483^

##### TR

Hypothyroidism-associated dyslipidemia promotes hepatic lipid accumulation, contributing to MASLD development and insulin resistance.^484^ Given the link between hypothyroidism and MASLD, increasing attention has been focused on TRβ, the predominant isoform in the liver.

TRβ promotes lipophagy, mitochondrial biogenesis, and mitophagy, thereby enhancing hepatic fatty acid β-oxidation and reducing lipotoxicity. It also facilitates LDL uptake and improves lipid profiles.^179^ The TRβ-selective agonist resmetirom has shown efficacy in reducing hepatic fat content, lowering liver enzyme levels, improving noninvasive fibrosis markers, and decreasing liver stiffness. Additionally, resmetirom confers cardiovascular benefits, including reductions in serum LDL cholesterol. Phase 3 trial data further demonstrate MASH resolution and fibrosis improvement after 52 weeks of treatment.^179^

##### PXR

Multiple variants of the NR1I2, the gene encoding PXR, have been identified in humans. Notably, at least two polymorphisms—rs7643645/G and rs2461823—are associated with increased MASLD severity.^485^ Nonetheless, PXR is anti-inflammatory and may reduce hepatic fibrogenesis, but PXR activation has been associated with obesity, insulin resistance, and hypercholesterolemia.^486^ Both activation and silencing of PXR induce lipid accumulation in human hepatocytes through distinct lipogenic pathways, as demonstrated *in vitro*.^487^ In animal models, PXR ablation suppresses HFD-induced obesity, steatosis, and insulin resistance,^488^ whereas PXR activation recapitulates key features of MASLD and MASH, including steatosis, inflammation, and lipotoxicity.^452^

##### HNF4α

HNF4α is highly expressed in the liver, and its expression is significantly reduced in both patients with MASLD and in MASH mouse models.^489^ HNF4α plays a critical role in MASLD progression.^489^^,^^490^ In hepatocytes, HNF4α prevents hepatic triglyceride accumulation by promoting triglyceride hydrolysis, FAO, and VLDL secretion. Overexpression of HNF4α ameliorates diet-induced steatohepatitis, whereas hepatocyte-specific deletion of HNF4α has the opposite effect. Notably, HNF4α has been shown to suppress the progression from MASLD to MASH.^491^^,^^492^

##### Other NRs in MASLD

Constitutive androstane receptor (CAR) activity increases with the severity of MASLD. CAR modulates hepatic metabolism in the fed state, reducing steatosis, inflammation, obesity, insulin resistance, and hypercholesterolemia.^486^ Administration of CAR agonists alleviate hepatic inflammation and hepatocyte apoptosis, supporting that CAR activation may be beneficial in mitigating steatohepatitis.^493^

REV-ERBα/β regulate circadian rhythm and coordinate lipid and glucose metabolism.^209^ They also modulate inflammatory responses during MASLD progression.^494^^,^^495^ REV-ERBα/β activation suppresses lipogenesis, improves dyslipidemia and hyperglycemia, and attenuates hepatic fibrosis and inflammation.^209^^,^^495^

#### NRs in obesity

Obesity is driven by complex mechanisms, including dyslipidemia, insulin resistance, and adipose inflammation. NRs contribute to these pathological processes and have emerged as key regulators of obesity.

##### PPARs

As lipid sensors, PPARs are pivotal lipid metabolic NRs actively involved in the control of obesity and its related metabolic disorders. Evidence suggests that PPARα and PPARβ/δ are potential therapeutic targets to prevent obesity,^496^ while PPARγ, a master regulator of adipogenesis, modulates obesity-related phenotypes.^497^ Pharmacological activation of PPARα has beneficial effects on glucose homeostasis, insulin resistance, inflammation, and hyperlipidemia.^498^^,^^499^^,^^500^ PPARα-deficient mice exhibit impaired brown adipose tissue development and reduced FAO and thermogenesis.^501^ In diet-induced obesity models, PPARα activation upregulates thermogenic genes and energy expenditure, contributing to weight loss.^502^

PPARβ/δ activation improves lipid homeostasis, prevents weight gain, and enhances insulin sensitivity.^503^ In animal models, PPARδ agonists reduce lipid accumulation, whereas PPARδ-deficient mice fed an HFD show reduced energy uncoupling and increased obesity susceptibility. *In vitro*, PPARδ activation promotes FAO in adipocytes and myocytes supporting its anti-obesity role.^504^

PPARγ is essential for adipocyte differentiation.^11^ Mice lacking PPARγ fail to develop adipose tissue.^505^^,^^506^ PPARγ induces adipocyte hypertrophy and insulin resistance in diet-induced obesity.^507^ Despite their efficacy in T2D, PPARγ agonists such as thiazolidinediones (TZDs) cause adverse effects like weight gain by stimulating lipogenic gene expression, including AP2, CD36, SCD-1, and SREBP-1.^496^

##### LXRs

LXRs serve as key metabolic regulators in obesity by modulating lipogenesis, maintaining cholesterol homeostasis, enhancing insulin sensitivity, and inhibiting inflammation. Despite their anti-atherogenic effects, LXR activation induces hepatic steatosis and hypertriglyceridemia via upregulation of lipogenic genes.^468^ In 3T3-L1 adipocytes, LXR agonist T0901317 significantly increases the expression of lipogenic genes such as FASN, ADD1/SREBP-1c, and PPARγ.^508^ Similarly, LXR activation promotes FASN-mediated lipogenesis through LPCAT3 induction, contributing to HFD-induced obesity and insulin resistance.^509^ LXRβ also implicates in adipose tissue inflammation and macrophage dysfunction during obesity, highlighting its potential as a therapeutic target.^510^ Notably, a liver-selective inverse agonist SR9243 has been shown to reduce inflammation, fibrosis, body weight, glucose, and lipid levels in MASH models.^221^

##### FXR

FXR is a central regulator of energy metabolism, maintaining glucose, lipid, and amino acid homeostasis,^511^ and has emerged as a promising therapeutic target for obesity-related metabolic disorders.^512^ FXR knockout mice exhibit elevated serum bile acids, hepatic and circulating triglycerides, cholesterol, and phospholipids.^513^ In animal models, treatment with the FXR agonist GW4064 alleviates diet-induced weight gain, prevents hepatic steatosis, and improves insulin sensitivity.^337^^,^^514^ Moreover, the gut-restricted FXR agonist fexaramine reduces diet-induced weight gain, systemic inflammation, and hepatic glucose production, while enhancing thermogenesis in white adipose tissue.^515^ These findings support FXR agonists as promising candidates for obesity-associated metabolic dysfunction.

##### VDR

VDR modulates adipose tissue metabolism, including adipocyte differentiation, lipogenesis, inflammation, and adipokine expression.^516^ VDR knockout mice display reduced fat mass, increased energy expenditure, and resistance to HFD-induced obesity, alongside improved glucose tolerance and insulin sensitivity.^517^^,^^518^ *In vitro*, VDR knockdown impairs adipogenesis in 3T3-L1 cells, whereas 1,25(OH)_2_D_3_ promotes differentiation of adipose-derived stem cells into adipocytes.^519^^,^^520^ Conversely, VDR overexpression in adipose tissue increases body weight and fat mass, disrupts glucose and lipid metabolism, induces insulin resistance, and impairs thermoregulation.^516^

##### TR

TRα primarily regulates thermogenesis, whereas TRβ predominantly controls cholesterol metabolism and lipogenesis.^11^ TRα-deficient mice are leaner than wild-type controls, with increased thermogenic energy expenditure, enhanced fat oxidation, and reduced susceptibility to HFD-induced obesity.^521^ Treatment with synthetic TRβ agonists KB-141 in ob/ob mice improves glucose tolerance and insulin sensitivity in a dose-dependent manner, supporting the potential of selective TRβ activation for anti-obesity, lipid-lowering, and antidiabetic therapy.^522^

##### ER

ERα and ERβ also influence body weight and fat distribution. ERα-deficient (ERα^−/−^) mice develop obesity with reduced energy expenditure, decreased physical activity, increased adiposity, impaired glucose homeostasis, and hyperleptinemia.^523^ Moreover, in ERα knockout mice, genes involved in hepatic lipid biosynthesis are upregulated, while lipid transport genes are downregulated. Notably, ablation of ERα, but not ERβ, leads to metabolic disturbances including weight gain, exacerbated adiposity, and impaired glucose tolerance.^524^^,^^525^ A recent study by Yang et al. demonstrated that an ERα-derived peptide improves insulin sensitivity and ameliorates glucose homeostasis and lipid profiles during obesity.^526^ Additionally, ER signaling may be implicated in fat distribution.^527^

##### AR

A study on AR knockout (ARKO) female mice showed increased body weight, elevated hepatic triglyceride, reduced insulin sensitivity, and higher plasma cholesterol levels, suggesting a protective role of AR against diet-induced obesity and dyslipidemia in females. Rubinow et al. further demonstrated that wild-type mice receiving ARKO bone marrow exhibited significantly greater visceral and total fat mass under HFD feeding compared to those receiving wild-type bone marrow.^528^

##### GR

GR and its downstream signaling cascade are key drivers of visceral adipocyte differentiation and metabolic dysfunction.^11^ Cushing’s syndrome, a classic example of glucocorticoid excess, is characterized by excessive visceral fat accumulation,^529^ supporting the role of GR in adipose-tissue homeostasis and fat redistribution.^530^ *In vitro* studies demonstrate that GR agonists promote adipocyte differentiation, whereas GR knockout impairs adipogenesis and improves glucose tolerance and insulin sensitivity.^531^^,^^532^

##### Other NRs in obesity

Mice lacking RXRα in adipocytes exhibit resistance to diet- and chemically induced obesity, along with impaired fasting-induced lipolysis.^533^ Pharmacological activation of RXR improves insulin sensitivity and attenuates hyperglycemia, hypertriglyceridemia, hyperinsulinemia, weight gain, and food intake in diabetic and obese mouse models.^11^

PXR deficiency protects against diet-induced obesity, insulin resistance, and hepatic steatosis.^488^ Conversely, PXR activation promotes hepatic triglyceride accumulation and contributes to steatosis.^534^ Treatment with CAR agonists alleviates hepatic steatosis in both HFD-fed and ob/ob mice, enhances insulin sensitivity, improves glucose and lipid metabolism, and prevents diet-induced obesity.^535^

#### NRs in cardiovascular diseases

Given their multiple roles in metabolic regulation and inflammation control, NRs are critically involved in the pathogenesis of various cardiovascular diseases.

##### Atherosclerosis

Atherosclerosis is a chronic inflammatory disease characterized by endothelial dysfunction, dysregulated lipid metabolism, and aberrant immune responses. NRs, particularly PPARs and LXRs, play central roles in the regulation of lipid metabolism, vascular inflammation, and remodeling during the development of atherosclerosis.

##### PPARs

PPARs modulate multiple stages of atherosclerosis, including endothelial dysfunction, macrophage foam cell formation, inflammation, and vascular smooth muscle cell (VSMC) proliferation and migration.^536^ Activation of PPARα and PPARγ reduces the secretion of endothelin-1 (ET-1), downregulates endothelial adhesion molecules,^537^^,^^538^ and suppresses NF-κB signaling.^539^^,^^540^ PPARα and PPARδ exert vasoprotective effects by upregulating endothelial nitric oxide synthase (eNOS) expression and enhancing nitric oxide (NO) production.^541^^,^^542^ Additionally, PPARγ mitigates oxidative stress by modulating the activity of intracellular antioxidant systems in endothelial cells.^543^^,^^544^

PPARs also play critical roles in macrophage foam cell formation and polarization. Although PPAR activation increases the expression of cholesterol uptake receptors such as CD36 and SR-A, its net effect is to promote cholesterol mobilization and efflux, thereby limiting intracellular cholesterol accumulation. This is primarily mediated through induction of cholesterol efflux transporters, including ABCA1, ABCG1, and SR-BI.^536^ PPARα attenuates macrophage inflammation by promoting polarization toward the anti-inflammatory M2 phenotype.^545^ PPARβ/δ deficiency impairs this M2 polarization.^283^ Similarly, PPARγ facilitates M2 polarization and suppresses M1 polarization, thereby contributing to an anti-inflammatory milieu.^346^

##### LXRs

LXRs exert anti-atherosclerotic effects through several mechanisms. LXR activation upregulates the expression of hepatic cholesterol transporters such as ABCA1 and ABCG1, promoting cholesterol efflux and lowering plasma cholesterol levels, thereby slowing atherosclerosis progression.^48^ Additionally, LXR induces the expression of the E3 ubiquitin ligase Idol, leading to the degradation of LDL receptors and inhibition of cholesterol uptake.^546^ LXR agonists reverse oxLDL-induced expression of VCAM-1 in endothelial cells, thereby reducing monocyte adhesion and migration.^547^

Moreover, LXRs suppress vascular inflammation by inhibiting NF-κB-dependent transcription of pro-inflammatory genes.^548^ Despite these protective effects, LXR agonist administration induces hepatic lipogenesis and elevates triglyceride levels, leading to steatosis in mice,^549^ an effect driven by LXRα-mediated upregulation of SREBP-1c and FASN expression.^44^

##### FXR

FXR reduces macrophage cholesterol uptake by modulating the expression of CD36 and ABCA1.^550^ FXR activation also suppresses ET-1, upregulates eNOS,^551^ inhibits plaque formation, lowers circulating lipid levels,^552^ and attenuates smooth muscle cells (SMCs) inflammation and migration.^553^ Conversely, FXR deficiency exacerbates LPS-induced production of IL-6 and TNF-α in macrophages^550^ and increases plaque burden in ApoE^−/−^ mice.^554^

##### NR4A

The NR4A subfamily acts as transcriptional regulators in metabolic and vascular diseases. NR4A are rapidly induced by pro-atherogenic stimuli.^555^ Nur77 and Nurr1 have been shown to confer atheroprotective effects, whereas NOR1 appears to promote disease progression.^150^^,^^556^ Nur77 suppresses oxLDL-induced inflammatory responses in macrophages by downregulating the expression of COX-2.^557^ Nur77 deficiency promotes M1 polarization and exacerbates atherosclerosis in LDLR^−/−^ and ApoE^−/−^ mice.^558^^,^^559^ In contrast, Nur77 induction reduces macrophage-derived foam cell formation and hepatic lipid accumulation and downregulates genes involved in inflammation, adhesion, and intestinal lipid absorption, ultimately decreasing plaque formation.^560^ Nurr1 exerts anti-proliferative and anti-inflammatory effects in SMCs^561^ and promotes M2 macrophage polarization by directly binding to the promoter of arginase-1 and activating its transcription.^562^ In contrast, NR4A3 appears pro-atherogenic. NR4A3 deficiency reduces vascular injury and atherosclerosis in mice.^563^^,^^564^ Zhao et al. showed that NOR1 promotes monocyte adhesion by inducing the expression of VCAM-1 and ICAM-1 in endothelial cells, while NOR1 deficiency reduces atherosclerotic lesion formation in ApoE^−/−^ mice by decreasing macrophage content in plaques.^565^ Consistent with these findings, our previous study demonstrated that glycated ApoA-IV induces pro-inflammatory response and promotes atherosclerosis in ApoE^−/−^ mice through NR4A3.^151^

##### Other NRs in atherosclerosis

PXR activation induces hypercholesterolemia, potentially through SREBP2-mediated upregulation of cholesterol biosynthetic genes, including the rate-limiting enzyme HMG-CoA reductase. Additionally, PXR increases hepatic PCSK9 expression, which leads to degradation of LDL receptors, reducing LDL clearance, and elevating circulating LDL levels.^566^ PXR deficiency reduces CD36 expression and lipid accumulation in macrophages, thereby attenuating atherosclerosis in ApoE^−/−^ mice.^567^

In our recent study, we reported a novel role for DAX1 in atherosclerosis. We found that DAX1 expression is upregulated in macrophages within atherosclerotic plaques. Macrophage-specific DAX1 deletion suppressed atherosclerosis progression. Mechanistically, DAX1 interacts with LXRα and TFEB to inhibit cholesterol transport and autophagy, resulting in lipid accumulation and inflammation in macrophages.^172^

##### Myocardial infarction

Myocardial infarction (MI) triggers a complex response involving inflammation, metabolic dysregulation, cell death, and tissue remodeling. NRs play critical roles in these processes by regulating gene expression related to inflammation, FAO, and cardiac repair.

##### PPARs

NRs play key roles in the metabolic regulation of cardiomyocytes. Among them, PPARs have been extensively studied in MI.^568^ Sambandan et al. demonstrated that chronic or excessive activation of PPARα is detrimental to post-ischemic cardiac recovery: cardiac-specific PPARα overexpression increased FAO, reduced glucose oxidation, and impaired functional recovery, whereas PPARα knockout hearts exhibited reduced FAO, enhanced glucose oxidation, and improved post-ischemic performance.^569^ Consistently, another study reported that cardiomyocyte-specific overexpression of PPARα exacerbates irreversible ischemic damage.^570^ However, pharmacologic activation of PPARα after MI has been shown to reduce infarct size and improve cardiac function, potentially by mitigating the decline in FAO enzyme activity, suppressing pro-inflammatory cytokine release and neutrophil infiltration, and inhibiting NF-κB activation.^571^^,^^572^ These findings indicate that the net effect of PPARα activation is context dependent—governed by timing, dose/exposure duration, and tissue selectivity.

PPARβ/δ is the predominant PPARs isoform in cardiac tissue^573^ and functions as a central node sustaining FAO and maintaining energy balance and normal cardiac function.^574^ Inducible, cardiomyocyte-specific activation or overexpression of PPARβ/δ after MI reduces infarct size, improves cardiac function, and promotes cardiomyocyte proliferation, indicating a protective role in the injured heart.^575^ Conversely, cardiomyocyte-restricted PPARβ/δ deletion decreases myocardial FAO, increases superoxide levels, and impairs cardiac structure and function.^574^^,^^576^

The role of PPARγ in MI has primarily been explored through pharmacological activation. TZDs (such as rosiglitazone and pioglitazone), PPARγ agonists, are widely used in T2D management, but their cardiovascular safety remains controversial due to serious adverse effects.^577^^,^^578^ Rosiglitazone has been associated with an increased risk of MI and stroke.^579^ However, animal models of ischemia/reperfusion have shown that treatment with rosiglitazone or pioglitazone reduces infarct size and improves systolic function.^580^^,^^581^^,^^582^ A recent study demonstrated that gabapentin, a γ-aminobutyric acid analog, attenuates post-infarction cardiac remodeling by suppressing M1 macrophage polarization via a PPARγ-dependent mechanism.^583^

##### MR

Cardiomyocyte-specific MR deficiency improves infarct healing and prevents the progression of ischemic heart failure (HF) by attenuating adverse cardiac remodeling, systolic dysfunction, and maladaptive molecular changes, highlighting the critical role of cardiomyocyte MR in cardiac function.^584^ A recent study demonstrated that MR deletion in T cells markedly reduces macrophage accumulation and pro-inflammatory macrophage polarization in the infarcted myocardium, thereby mitigating MI severity.^585^ MRAs have been shown to improve outcomes in patients following MI.^586^^,^^587^ Early administration with MRAs after MI enhances infarct healing and attenuates both electrical and structural cardiac remodeling.^588^

##### Other NRs in MI

Several other NRs have been implicated in MI. LXRα acts as an endogenous cardioprotective factor in ischemic heart disease.^216^ LXRα deficiency impairs glucose uptake after MI, leading to metabolic shifts, reduced glycolysis, and adverse cardiac remodeling.^589^

ERβ has also been identified as cardioprotective in ischemic injury.^590^^,^^591^^,^^592^ ERβ activation improves MI-induced cardiac dysfunction by enhancing Notch1 signaling.^593^ GR ablation or pharmacological antagonism have been shown to enhance cardiomyocyte survival, cell-cycle re-entry, and mitosis, thereby promoting myocardial regeneration and reducing scar formation.^594^^,^^595^ GR antagonism has emerged as a potential strategy to stimulate endogenous cardiomyocyte proliferation and promote myocardial regeneration after cardiac injury.^594^^,^^595^^,^^596^

Reduced expression of RXRα after MI is associated with increased infarct size at later stages. In mononuclear phagocytes, decreased RXRα expression impairs phagocytic activity, leading to the accumulation of apoptotic cells in cardiac tissue, reduced angiogenesis in the infarct border zone, suppressed macrophage proliferation, and altered monocyte/macrophage subset composition.^597^ Activation of NR4A1 mitigates cardiac fibrosis post-MI by modulating glycolysis through regulation of ENO1.^598^ Conversely, NR4A1 deficiency increases CCR2 expression in Ly-6C^high^ monocytes and promotes myocardial infiltration and macrophage polarization toward a hyperinflammatory phenotype, ultimately impairing cardiac healing and function.^599^ NR4A2 is upregulated in the infarcted heart, and it mitigates post-MI injury and adverse remodeling by promoting autophagy and inhibiting apoptosis.^600^

##### HF

HF is a complex clinical syndrome involving cardiomyocyte injury, metabolic dysregulation, mitochondrial dysfunction, inflammation, oxidative stress, and pathological remodeling characterized by hypertrophy, fibrosis, and apoptosis. Increasing evidence highlights the pivotal role of NRs in the pathogenesis and progression of HF.

##### MR

MR plays a critical role in the development of HF. Its overactivation contributes to coronary endothelial dysfunction, cardiomyocyte apoptosis, and reactive myocardial fibrosis, ultimately promoting adverse cardiac remodeling. MRAs have demonstrated clinical benefits in improving outcomes in HF.^601^ Spironolactone and eplerenone, two widely used MRAs, have been shown to significantly reduce cardiovascular morbidity and mortality in patients with chronic HF and reduced ejection fraction (HFrEF).^602^^,^^603^ In experimental HF models, MR blockade decreases the expression of galectin-3 (Gal-3) and soluble ST2 (sST2) without interfering with IL-33-mediated cardioprotection, which are associated with the reduction of myocardial fibrosis and inflammation.^604^

##### PPARs

As HF progresses, ATP depletion impairs myocardial energy metabolism, largely due to suppressed FAO, which is associated with deactivation of the transcriptional coregulators PPARgamma coactivator 1 (PGC-1)/PPAR signaling.^605^ In animal models of HF, PPARα activation enhances FAO, improves endothelial function, and reduces myocardial fibrosis and hypertrophy.^606^ PPARα agonist fenofibrate attenuates cardiac fibrosis, lipid accumulation, and inflammation by modulating TNF signaling, and its use has been linked to a reduced risk of HF-related hospitalization.^607^

Cardiomyocyte-specific deletion of PPARβ/δ results in cardiac dysfunction, progressive myocardial lipid accumulation, hypertrophy, and congestive HF with reduced survival, likely due to impaired FAO.^574^ PPARδ activation restores stromal metabolism, attenuates right ventricular hypertrophy,^608^ and suppresses cardiac fibroblast proliferation and myofibroblast transdifferentiation.^609^

PPARγ agonist pioglitazone reverses pulmonary hypertension and prevents right ventricular failure by enhancing FAO.^610^ PPARγ activation also exerts antifibrotic effects by inhibiting angiotensin II-induced proliferation and differentiation of cardiac fibroblasts,^611^^,^^612^ whereas PPARγ inhibition promotes fibroblast activation and differentiation.^613^^,^^614^

##### Estrogen-related receptors

Estrogen-related receptors (ERRs) are important regulators of cardiac energy metabolism. PGC-1 acts with ERRs to drive mitochondrial biogenesis and energy transduction pathways, including the tricarboxylic acid cycle and FAO. During HF progression, metabolic and contractile programs shift: the capacity for FAO and mitochondrial respiration declines, in part due to deactivation of the PGC-1/ERR signaling.^605^ Xu et al. demonstrated that activation of ERRs ameliorates HF by enhancing enhanced FAO and mitochondrial function.^111^

##### NR4A

Among the three NR4A family members, Nur77 is abundantly expressed in the heart and is rapidly upregulated in response to various stress stimuli, including isoproterenol, angiotensin II, transverse aortic constriction (TAC), ischemia/reperfusion injury, and MI.^153^ Nur77 deficiency results in altered cardiac Ca^2+^ homeostasis and cardiac remodeling.^615^ Liu et al. proposed that NR4A2’s autophagy-enhancing and anti-apoptotic actions support its candidacy as a therapeutic target for HF.^600^ In contrast, NOR1 contributes to cardiac hypertrophy and adverse remodeling.^616^^,^^617^

##### Other NRs in HF

Other NRs have also been implicated in the pathogenesis of HF. During the compensatory phase of HF, TRα1 expression is upregulated but declines with progression to decompensated left ventricular dysfunction.^618^ In ischemic heart models, early and sustained restoration of T3 levels prevents the progression of HF, potentially through enhanced angiogenesis, mitochondrial biogenesis, Ca^2+^ transients and contractility, and decreased fibrosis.^619^^,^^620^ Cardiomyocyte-specific GR deletion leads to spontaneous cardiac hypertrophy, impaired systolic function, and premature death from HF, underscoring the critical role of GR signaling in maintaining cardiac structure and function.^621^ VDR polymorphisms, particularly FokI and TaqI, have been associated with HF risk. Combined analysis of serum vitamin D levels and VDR genetic variants suggests a link between vitamin D deficiency, VDR mutations, and susceptibility to chronic HF.^622^^,^^623^

##### Abdominal aortic aneurysm

Abdominal aortic aneurysm (AAA) is characterized by structural deterioration of the aortic wall, driven by persistent infiltration of inflammatory cells, primarily macrophages, and lymphocytes, along with enhanced activity of proteolytic enzymes, particularly matrix metalloproteinases (MMPs). These changes contribute to the degradation of elastin and collagen, extensive VSMC apoptosis and phenotypic switching, and oxidative stress-induced injury. NRs have emerged as important regulators of AAA development by modulating these pathological processes.

##### PPARs

PPARα activation reduces AAA incidence in ApoE^−/−^ and LDLR^−/−^ mice, by downregulating osteopontin, MMP-9, and resistin, limiting macrophage recruitment to the aortic wall and suppressing extracellular matrix remodeling.^624^^,^^625^ However, clinical studies have not shown clear benefits of the PPARα agonist fenofibrate on AAA biomarkers or aneurysm growth.^626^^,^^627^ PPARβ/δ activation attenuates angiotensin II-induced AAA formation in ApoE^−/−^ mice by reducing macrophage infiltration, preventing SMC loss, and downregulating monocyte chemoattractant protein-1 (MCP-1) and MMP-2 expression.^628^ PPARγ activation also limits AAA progression and rupture by suppressing aortic inflammation and macrophage infiltration.^624^^,^^629^ These findings are supported by clinical observations showing that treatment with the PPARγ agonist pioglitazone reduces inflammation in AAA patients, evidenced by decreased macrophage infiltration in the aortic wall and periaortic adipose tissue, along with reduced expression of TNF-α and MMP-9.^630^ Collectively, these studies suggest that PPAR activation may offer therapeutic benefits in the treatment of AAA.

##### ER and AR

Given the male predominance of AAA,^631^^,^^632^ ER and AR have been investigated for their roles in AAA pathogenesis. 17β-estradiol attenuates angiotensin II-induced AAA formation in ApoE^−/−^ mice, which is associated with the suppression of pro-inflammatory gene expression in the aorta, including ICAM-1, VCAM-1, and MCP-1.^632^^,^^633^ Tamoxifen, a selective estrogen receptor modulator (SERM), also inhibits AAA progression by reducing oxidative stress and neutrophil infiltration.^634^ Conversely, expression is upregulated in human AAA tissues.^635^ AR-deficient (ARKO) mice exhibit decreased aortic expression of pro-inflammatory cytokines such as Il-1α, Il-6, Ifnγ, and Il-17, accompanied by attenuated AAA growth,^636^ whereas pharmacological activation of AR promotes AAA formation.^637^

##### MR

A 25-year clinical follow-up study demonstrated an association between MR antagonism and attenuated AAA progression.^638^ Animal studies support this, showing that treatment with MRAs suppress inflammatory gene expression (Tnf-α, Il-6, Mcp-1, Mmp-2) and macrophage infiltration in the aortic wall and perivascular adipose tissue and reduce AAA formation.^639^ In contrast, administration of MR agonists in the presence of high salt promoted aortic aneurysm formation and rupture.^640^ However, another study reported that aldosterone infusion did not affect AAA development in ApoE^−/−^ mice.^641^

##### Other NRs in AAA

A growing number of animal studies have demonstrated the involvement of additional NRs in AAA pathogenesis. VDR activation reduces macrophage infiltration, neovascularization, and production of endothelial proinflammatory and angiogenic chemokines, thereby inhibiting angiotensin II-induced dissecting AAA formation in ApoE^−/−^ mice.^642^ Nur77 expression is reduced in human and mice AAA lesions. Its deletion accelerated the AAA development, which is associated with impaired anti-inflammatory regulation via suppression of LOX-1 signaling.^643^ In contrast, vascular overexpression of NOR1 enhances the aortic response to angiotensin II, promoting aneurysm formation in mice.^644^

NR1D1 (REV-ERBα) is significantly upregulated in AAA tissues and contributes to disease progression by suppressing mitochondrial metabolic enzymes such as ACO2, thereby impairing energy homeostasis and increasing oxidative stress.^37^ As a key metabolic regulator, LXRα was recently shown to be upregulated in both human and murine AAA tissues. VSMC-specific deletion of LXRα alleviated AAA formation by reducing extracellular matrix degradation, inflammation, and VSMC phenotypic switching.^645^ In addition, PXR has been shown to inhibit AAA by suppressing oxidative stress.^646^

##### Vascular and valvular calcification

NRs are closely involved in the pathogenesis of vascular and valvular calcification. Calcification is now recognized not as a passive mineral deposition but as an actively regulated process governed by multiple signaling pathways. In response to pathological stimuli, including hyperphosphatemia, inflammatory cytokines, oxidative stress, and mechanical stress, VSMCs and valvular interstitial cells undergo phenotypic switching toward osteogenic or chondrogenic-like states, ultimately leading to hydroxyapatite deposition within the vascular wall or valvular tissue.^647^ NRs act as cellular sensors of endocrine, metabolic, and inflammatory signals, modulating both cellular behavior and the local microenvironment.

##### VDR

VDR is a central component of the vitamin D signaling and plays a key role in calcium and phosphate homeostasis. At pharmacologic exposures that raise serum calcium and phosphate, VDR activators have been linked to increased vascular calcification, whereas at lower and protective dosages, they can protect against aortic calcification.^648^ In VDR^−/−^ mice, high-dose vitamin D3 failed to induce vascular calcification, indicating that VDR is required for vitamin D3-induced calcification.^649^ Interestingly, in an aortic transplantation model, transplanting VDR^−/−^ aortas into wild-type recipients followed by uremia induction and calcitriol treatment showed no difference in calcification between VDR^−/−^ allografts and adjacent VDR^+/+^ aorta, suggesting that VDR activation promotes calcification through systemic rather than direct vascular actions.^650^ However, VDR^−/−^ mice develop enhanced aortic root calcification, accompanied by upregulation of osteoblastic differentiation factors such as muscle segment homeobox (Msx2), bone morphogenetic protein 2 (BMP2), and runt-related transcription factor 2 (Runx2).^651^ Collectively, VDR’s role in calcification is context dependent, and conditional VDR knockout or overexpression approaches will be helpful to delineate its effects and mechanisms.

##### PPARs

Among the PPAR isoforms, PPARγ has been extensively studied in vascular calcification. VSMC-specific PPARγ deficiency accelerates vascular calcification, largely due to loss of PPARγ-mediated suppression of Wnt5a signaling, which activates the chondrogenic program.^652^ The PPARγ agonist pioglitazone attenuates aortic valve calcification by downregulating RAGE, reducing lipid deposition, calcium deposits, and apoptosis within the valve tissue.^653^^,^^654^ In rat aortic VSMCs, pioglitazone also inhibits β-glycerophosphate-induced calcification via suppression of the Wnt/β-catenin pathway.^655^ A recent study showed that PPARγ suppresses vascular calcification by inducing Klotho, a direct transcriptional target of PPARγ that is functionally linked to vascular mineral metabolism.^656^ In cultured VSMCs, PPARγ activation reduces the expression of osteogenic markers, including osteocalcin, BMP2, and core-binding factor α1 (Cbfα1), thereby attenuating high glucose-induced calcification.^657^ Several other studies consistently support a protective role of PPARγ signaling in vascular calcification.^658^^,^^659^ In addition, PPARα activation has been shown to alleviate hyperlipidemia-induced vascular calcification by suppressing autophagy-dependent ferroptosis triggered by mitochondrial DNA stress.^660^

##### LXR

*In vitro* studies have demonstrated that LXR activation enhances PKA-induced vascular calcification,^661^ whereas LXR inhibition attenuates this effect, potentially due to reduced LXR-mediated lipogenesis.^662^ A recent study showed that macrophage-targeted delivery of the LXR agonist T0901317 via hydrogel encapsulation significantly reduced arterial calcification without inducing hepatic steatosis.^383^ Additionally, LXR activation alters calcium and lipid deposition in cardiac valves, potentially through modulating inflammatory signaling.^663^

##### MR

MR activation upregulates calcification-associated genes such as TNAP/ALP and BMP-2 in VSMCs, thereby promoting osteogenic differentiation and calcification.^664^ The MRA spironolactone has been shown to prevent VC in both chronic kidney disease (CKD) rats and Klotho-deficient mice, likely through attenuation of MR signaling, local inflammation, osteogenic transdifferentiation, and apoptosis.^665^

##### NR4A

NR4A1-mediated mitochondrial fission and BNIP3-dependent mitophagy are implicated in lactate-induced acceleration of vascular calcification.^666^ Our recent findings demonstrated that NR4A3 is upregulated in murine models of CKD and 1,25(OH)_2_D_3_ overload, as well as in calcified human aortic tissue. NR4A3 deficiency preserves the contractile VSMC phenotype, suppresses osteogenic markers expression, and reduces calcium deposition, mechanistically through NR4A3-mediated glycolysis and histone lactylation.^152^ Although studies on NR4A2 in vascular calcification are limited, a prospective population-based study reported that NR4A2 haplotypes may be associated with increased aortic and coronary artery calcification, although the underlying mechanisms remain unclear.^667^

##### FXR

FXR activation attenuates vascular calcification in ApoE^−/−^ mice with CKD.^668^ Consistently, a recent study demonstrated that FXR activation suppresses TGFBR1/TAK1 signaling via miR-135a-5p to inhibit inflammation and osteogenic differentiation of SMCs, thereby attenuating vascular calcification in CKD rats.^669^ In contrast, FXR deficiency causes vascular calcification.^670^ These findings support FXR as a promising therapeutic target in vascular calcification.

##### Other NRs in vascular and valvular calcification

Studies have shown that antagonism or silencing of ERα or ERβ promotes the osteogenic differentiation and calcification of VSMCs *in vitro*.^671^ ERRγ contributes to vascular calcification by upregulating the BMP2 signaling pathway, while its inhibition reduces osteogenic gene expression and calcification *in vitro* and *in vivo*.^672^ Moreover, VSMC-specific AR ablation mitigates testosterone-induced vascular calcification.^673^ Activation of RAR inhibits calcification in primary human cardiovascular cells, whereas treatment with the RAR inhibitor AGN 193109 or small interfering RNA promotes calcification.^674^ RARα activation also prevents calcification of native aortic valves and bioprosthetic valves.^675^ In addition, macrophage-specific GR inactivation reduces vascular calcification in LDL receptor–deficient mice without altering atherosclerotic lesion size.^676^

##### Angiogenesis

NRs regulate angiogenesis by modulating endothelial function, vascular remodeling, and pro-angiogenic signaling. Their effects are context dependent, with different NRs exerting pro- or anti-angiogenic actions under various physiological and pathological conditions.

##### PPARs

PPARs are involved in the regulation of angiogenesis. The role of PPARα in angiogenesis appears context dependent, with both pro- and anti-angiogenic effects reported. These divergent outcomes likely depend on the tissue type, microenvironment, and receptor activation level.^677^ Studies using selective non-fibrate PPARα agonists or PPARα-deficient mice suggest a pro-angiogenic role for PPARα, potentially mediated by VEGF-dependent pathways.^678^ Notably, iloprost-induced angiogenesis and VEGF expression were abolished in PPARα^−/−^ mice, indicating a requirement for PPARα in this process.^679^ Conversely, other evidence supports an anti-angiogenic role of PPARα via inhibition of Akt activation, COX-2, prostaglandin E_2_, MMP-9, and VEGF, along with upregulation of endostatin and thrombospondin-1.^680^

The role of PPARγ in angiogenesis also remains controversial. Some *in vivo* studies establish angiogenic effects of PPARγ. Selective activation of PPARγ has been shown to stimulate neovascularization in the corneal angiogenic model via VEGF-dependent mechanism.^678^ PPARγ agonists restore angiogenesis in hindlimb ischemia and focal cerebral ischemia. Consistently, endothelial-specific PPARγ deletion impairs angiogenesis.^681^ However, other reports describe antiangiogenic actions of PPARγ activation, including enhanced endothelial NO production and maxi-K channel activation,^682^ inhibition of PKCα-mediated CREB activation and COX-2 expression,^683^ and downregulation of VEGF and VEGFR-2.^684^

PPARβ/δ activation consistently induces pro-angiogenic responses both *in vitro* and *in vivo*,^685^^,^^686^^,^^687^ potentially through modulation of pro-angiogenic targets such as VEGF, chloride intracellular channel protein 4, calcineurin, and platelet-derived growth factor receptor β.^680^

##### NR4A

Among the NR4A family, Nur77 has been extensively studied in angiogenesis. Several studies reported that Nur77 promotes endothelial cell proliferation, migration, and tube formation *in vitro*.^688^^,^^689^^,^^690^ In Nur77-deficient mice, VEGF-, histamine-, or serotonin-induced tumor growth; angiogenesis; and microvascular permeability are nearly abolished, suggesting that Nur77 may serve as a potential therapeutic target for tumors.^691^^,^^692^^,^^693^ Mechanistically, Nur77 regulates angiogenesis by modulating the expression of eNOS, VE-cadherin-associated junction components, and integrins.^693^^,^^694^

Our recent findings demonstrated that NR4A2 participates in MoS_2_NDs-induced autophagy activation, which enhances collateral vessel formation in ischemic diabetic mice.^695^ Additionally, NOR1 transcriptionally regulates ET-1 to mediate its pro-angiogenic effects,^696^ whereas antisense oligonucleotide-mediated knockdown of NOR1 reduces VEGF-induced endothelial proliferation and migration.^697^

##### ERRs

ERRγ has been identified as a hypoxia-independent inducer of neovascularization, promoting reparative revascularization.^698^ ERRα expression is upregulated in ischemic skeletal muscle of mice, and its overexpression enhances capillary, arteriole, and non-leaky vessel formation.^699^ In contrast, endothelial ERRα plays a repressive role in angiogenesis: ERRα downregulates the expression of angiogenesis-related genes, and its deletion in endothelial cells increased migration, sprouting, and tube formation.^700^

##### Other NRs in angiogenesis

RARα activation promotes endothelial cell proliferation and angiogenesis via mitogenic induction of FGF2.^701^ Endothelial GR deficiency enhances autophagic flux, leading to activation of Wnt/β-catenin signaling and promoting angiogenesis.^702^ MR negatively regulates angiogenesis by suppressing STAT3 activity in endothelial cells, suggesting that endothelial MR may serve as a therapeutic target to enhance neovascularization under ischemic conditions.^703^ Additionally, RORα modulates pathological retinal angiogenesis through SOCS3-dependent inflammatory pathways.^704^

##### Raynaud’s disease

Beyond the structural vascular pathologies reviewed above (atherosclerosis, AAA, and vascular calcification), a distinct category of functional vasomotor dysregulation—occurring *de novo* or secondary to structural disease—is exemplified by Raynaud’s disease. Experimental and clinical evidence indicates that ER pathways contribute to the exaggerated vasomotor responses that characterize Raynaud’s disease, with female predominance aligning with ER-dependent control of microvascular tone.^109^^,^^110^ Studies from Flavahan and Eid show that estrogenic signaling—via nuclear ERs and GPER—upregulates and mobilizes α2C-adrenergic receptors in cutaneous vascular smooth muscle, thereby potentiating cold-induced vasoconstriction.^705^^,^^706^^,^^707^^,^^708^ Mechanistically, these works implicate rapid, non-genomic cascades (notably cAMP-Epac-JNK-AP-1) that increase α2C-AR signaling capacity and shift the contractile set-point during cold exposure.^706^^,^^707^^,^^708^ Accordingly, it has been proposed that the ER-GPER-α2C axis provides a coherent explanation for cold sensitivity and sex bias in Raynaud’s disease and can serve as a therapeutic target.^109^^,^^708^

#### NRs in inflammation and immune diseases

NRs play central roles in chronic inflammatory and immune-mediated diseases, including inflammatory bowel disease (IBD), rheumatoid arthritis (RA), systemic lupus erythematosus (SLE), and MS. NRs influence disease progression through transcriptional control of cytokine production, immune cell differentiation, epithelial integrity, and tissue remodeling, and several have emerged as promising therapeutic targets.

In IBD, FXR signaling is downregulated in chronic intestinal inflammation, while its activation alleviates intestinal inflammation.^244^^,^^709^^,^^710^ Genetic variants of NR1H4 (such as rs3863377 and rs56163822) are associated with IBD susceptibility.^711^ VDR maintains epithelial barrier integrity and suppresses NF-κB signaling,^712^ whereas its deficiency exacerbates colitis.^713^ RXR agonists CBt-PMN ameliorate colitis by reducing pro-inflammatory cytokines such as Tnf and Il6.^81^ ERβ expression is reduced in active ulcerative colitis and Crohn’s disease,^714^^,^^715^ and epithelial ERβ deletion alters gut microbiota.^716^ Selective glucocorticoid receptor agonists exert anti-inflammatory effects comparable to conventional glucocorticoids without impairing mucosal healing.^717^ NR4A1 protects against colonic fibrosis by restraining TGF-β1-driven fibroblast activation.^272^ Other NRs, including CAR, LXR, PPARs, RAR, HNF4α, and NR2F6, regulate intestinal cell types, tight junctions, autophagy, and immune homeostasis.^718^

The LXR inverse agonist SR9243 alleviates RA by modulating glycolytic metabolism in macrophages.^719^ GR is overexpressed in the synovium of some RA patients and correlates with pro-inflammatory gene expression.^720^ The first clinical use of glucocorticoids, GR agonist, was for the treatment of RA. In RA models with GR deleted in myeloid cells, dendritic cells, B cells, or T cells, only T-cell-specific GR deletion attenuated the therapeutic benefit of glucocorticoids, indicating that T-cell-intrinsic GR signaling is necessary for anti-inflammatory efficacy.^721^ VDR SNPs are linked to RA risk.^722^ VDR deficiency enhanced inflammation, cartilage damage, and bone erosion.^723^ PPARγ is downregulated in RA synovial tissue^724^ and proposed as a biomarker for RA diagnosis and disease activity.^725^ NR4A2 is upregulated in RA synovium^726^ and promotes invasive synoviocyte phenotypes and cartilage destruction.^727^ Additionally, ERRα is dysregulated in inflammatory arthritis and contributes to RA-associated bone loss.^728^

In SLE, dexamethasone liposome-integrated mesenchymal stem cells (Dexlip-MSCs) activate GR signaling to upregulate cysteine-rich secretory protein LCCL-containing domain 2 (CRISPLD2) and suppress inflammatory mediators.^729^ RORα is reduced in SLE aortas and endothelial cells; melatonin restores RORα to preserve endothelial function.^730^ NR4A1 deficiency impairs regulatory T cell function and promotes inflammatory T cell activation, exacerbating autoimmunity and contributing to SLE pathogenesis, and neuronal restoration of NR4A1 protects against synaptic loss and neuropsychiatric symptoms in lupus-prone mice.^731^ ERα is upregulated in peripheral blood mononuclear cells and T cells of SLE patients, while ERβ levels are inversely correlated with SLE activity.^732^ Reduced VDR expression^733^ and VDR polymorphisms are associated with increased SLE risk, especially in Asian populations.^734^

In MS, VDR polymorphisms affect remyelination and disease susceptibility.^735^ GR signaling mitigates acute MS inflammation by expanding myeloid-derived suppressor cells via S100A8/A9.^736^ LXR suppresses MS-associated neuroinflammation via inhibition of NF-κB and NLRP3 inflammasome signaling^737^ and promotes remyelination and alleviates neurodegeneration,^738^ while NR1H3 mutations are linked to familial MS.^739^ RXR is essential for oligodendrocyte differentiation and remyelination,^740^ and its activation improves MS pathology.^741^ ERβ contributes to neuroprotection,^742^ and estrogen-based therapies reduce relapse rates.^743^ PPARα activation ameliorates MS-related neuropathology by modulating oxidative stress, autophagy, mitochondrial dysfunction, and inflammatory signaling.^744^ NR4A2, upregulated by inflammation, drives neurodegeneration via glycolytic and secretory pathways.^745^

### NRs as therapeutic targets

Given the central roles of NRs in metabolic homeostasis and inflammatory signaling, they have emerged as attractive therapeutic targets for metabolic and immune-mediated diseases. Pharmacological modulation NRs offers promising intervention strategies, and many NR-targeting ligands are clinically approved or under active investigation (Table 2).

#### TRs

TR-targeted therapies have traditionally been used to treat disorders directly related to TH function. However, advances in understanding the tissue-specific roles of TR isoforms, particularly TRβ, have expanded their potential application to non-classical indications, including MASH, dyslipidemia, demyelinating diseases, and certain cancer.^178^

Resmetirom is a selective TRβ agonist that regulates hepatic triglyceride and cholesterol metabolism, leading to reduced intrahepatic lipid content. Patients treated with resmetirom have shown significant decreases in liver fat and a higher rate of MASH resolution on biopsy. Moreover, treatment with resmetirom reduces alanine aminotransferase (ALT), γ-glutamyl transferase, serum fibrosis markers, and liver stiffness.^746^

#### RAR

RAR agonists have demonstrated efficacy across a range of conditions. All-trans retinoic acid, a pan-RAR agonist, is primarily used in acute promyelocytic leukemia to induce leukemic cell differentiation and is widely applied in acne treatment. Tazarotene, a RAR-selective agonist, is approved for psoriasis and acne.^747^^,^^748^ Recently, tectorigenin, a non-retinoid RARγ-selective agonist, has been shown to suppress UV-induced skin damage, suggesting its potential in photoaging and cutaneous inflammation.^191^

#### PPARs

PPAR agonists have shown efficacy in the treatment of T2D, MASLD, dyslipidemia, and cardiovascular diseases, but their clinical application is limited by adverse effects—such as weight gain and cardiovascular risks—and insufficient subtype selectivity.^749^ To address these limitations, selective PPAR modulators (SPPARMs) have been developed to improve subtype specificity and gene-targeting precision, while minimizing side effects. For instance, the SPPARMγ agent INT131 improves glycemic control with reduced weight gain.^203^ The PPARβ/δ agonist GW501516 was investigated for metabolic syndrome and diabetes but discontinued due to carcinogenicity in rodent models.^750^ Novel dual (such as saroglitazar) and pan-PPAR agonists (such as lanifibranor) aim to balance PPARα/γ/δ activity to optimize metabolic benefits and reduce adverse effects. Saroglitazar treatment reduces hepatic stiffness and improves metabolic parameters in patients with MASLD or MASH.^751^ A phase 3 trial of lanifibranor (NCT04849728) is underway in MASH patients with F2-F3 fibrosis.^746^

Several natural compounds also exhibit therapeutic potential via PPARγ activation. Forsythoside A alleviates acute lung injury by inhibiting inflammation and epithelial barrier disruption through the PPARγ/RXRα complex.^752^ 1,8-cineole improves diabetic retinopathy by suppressing ferroptosis in retinal pigment epithelial cells via the PPARγ/TXNIP pathway.^753^ Other agents—such as curcumin, apigenin, the traditional Chinese medicine Qiliqiangxin, and chrysin and hesperetin—may exert cardioprotective effects in myocardial ischemia through PPARγ activation.^568^

#### LXRs

Synthetic LXR agonists, such as T0901317 and GW3965, have shown therapeutic benefits in animal models of diabetes and atherosclerosis.^211^^,^^212^^,^^214^^,^^215^ However, their clinical translation has been limited by adverse effects, including hypertriglyceridemia and hepatic steatosis.^43^ Strategies that retain the metabolic benefits of LXR activation while minimizing lipogenic side effects are under active exploration. Recent efforts have focused on LXR inverse agonists as potential treatments for metabolic-associated fatty liver disease (MAFLD), MASH, and related disorders. These compounds recruit corepressors to suppress genes involved in *de novo* lipogenesis. Notable examples include SR9238 and SR9243, which have shown efficacy in preclinical models of MAFLD, dyslipidemia, and cancer.^219^^,^^221^ TLC-2716 is undergoing clinical evaluation for the treatment of severe dyslipidemia (NCT05483998).^42^

In addition, several natural compounds, such as kaempferol, 1,8-cineole, squalene, and resveratrol, activate the LXR signaling and exert therapeutic effects in MASLD. Conversely, natural inhibitors of LXR transcriptional activity, including alpinetin, morin, luteolin, and the hexane fraction of *Cyperus rotundus*, have shown protective effects against hepatic steatosis. These agents modulate LXR activity with fewer adverse effects, making them promising candidates for the treatment of metabolic liver diseases.^754^

#### FXR

FXR has emerged as a therapeutic target for diverse conditions, including primary biliary cholangitis, MASH, T2D, dyslipidemia, and IBD. FXR agonists—particularly OCA—have gained clinical use, while newer non-bile acid agonists with improved pharmacological properties, including cilofexor and tropifexor, are in development.^50^^,^^224^^,^^225^^,^^226^

In preclinical studies, the synthetic FXR agonist GW4064 reduced hepatic steatosis and limited weight gain by downregulating hepatic CD36 expression.^337^ Natural FXR activators, including curcumin and ethanol extracts of *Schisandra chinensis* fruit have shown benefits in hyperlipidemia, obesity, elevated liver enzymes, and hepatic steatosis.^755^^,^^756^

Conversely, intestinal FXR antagonists such as glycoursodeoxycholic acid (GUDCA) and glycine-β-muricholic acid (glycine-β-MCA) have been shown to modulate bile acid, lipid, and glucose metabolism, thereby alleviating obesity, insulin resistance, and hepatic steatosis.^233^^,^^234^

#### ER

ER signaling is closely associated with multiple diseases, notably hormone-dependent cancers (such as breast cancer), osteoporosis, and cardiovascular diseases. Pharmacological modulation of ER has become a key therapeutic strategy. SERMs, such as tamoxifen, antagonize ERs in breast tissue to inhibit estrogen-driven proliferation, while exerting estrogenic effects in other tissues (such as bone).^242^ Selective estrogen receptor degraders, including fulvestrant and elacestrant, bind to ERs and induce receptor degradation, offering effective treatment for ER-positive breast cancer.^757^^,^^758^ Beyond oncology, ER signaling confers cardiovascular protection, including mitigation of diabetes-induced vascular injury^759^ and attenuation of hepatic fibrosis.^243^ Notably, astragaloside IV has been shown to inhibit vascular calcification via ERα activation.^760^

#### GR

Synthetic glucocorticoids, such as prednisone,^761^ dexamethasone,^762^ and methylprednisolone,^763^ are widely used in the treatment of RA, SLE, IBD, and acute MS.^764^ Their anti-inflammatory effects are mainly achieved by suppressing NF-κB and AP-1 and inducing anti-inflammatory gene expression, resulting in broad immunosuppression. However, long-term glucocorticoids use is limited by severe adverse effects, including hyperglycemia, osteoporosis, muscle atrophy, and increased infection risk,^765^ prompting the development of selective glucocorticoid receptor modulators (SGRMs).^766^ Highly selective SGRMs, CORT118335 and CORT108297, have shown efficacy in preclinical models of MASLD and obesity.^118^^,^^254^^,^^255^

#### MR

Steroidal MRAs, including spironolactone and eplerenone, have been shown to reduce cardiovascular morbidity and mortality in patients with chronic HFrEF.^601^ However, their use is limited by the risk of serious adverse effects, notably hyperkalemia and renal impairment.^602^^,^^603^

Compared with steroidal MRAs, novel non-steroidal MRAs exhibit distinct pharmacological properties and improved safety profiles. Clinical trials have demonstrated their efficacy in hypertension and HF, particularly in patients with CKD and T2D, where they offer a safer alternative to conventional MRAs.^262^^,^^264^^,^^265^^,^^266^^,^^267^ Moreover, preclinical studies show that the novel MR modulator AZD9977 confers cardio-renal protection with minimal impact on electrolyte excretion, highlighting its therapeutic potential.^432^

#### VDR

VDR agonists have been widely studied in osteoporosis, autoimmune disorders, and cancer. Supplementation with vitamin D or its synthetic analog alfacalcidol significantly improves bone mineral density in osteoporosis,^236^^,^^237^ while paricalcitol has shown efficacy in diabetic nephropathy.^61^

#### Other NRs

Emerging novel agonists or antagonists targeting other NRs are also under investigation. The natural NR4A1 agonist cytosporone B (Csn-B) exhibits therapeutic potential in acute transplant rejection,^273^ MS,^274^ and fibrosis in tissues such as liver and gut.^272^ Additionally, our recent study identified 2′-deoxycytidine as a DAX1 inhibitor that effectively attenuates atherosclerosis.^172^

### Translational considerations

Despite extensive preclinical evidence supporting NR modulators in metabolic and cardiovascular diseases, clinical translation has faced substantial challenges. Several factors contribute to the gaps between bench and bedside.

First, species-specific differences often limit extrapolation, as illustrated by LXR agonists (such as BMS-852927) that reduced atherosclerosis in mice but raised triglycerides and reduced neutrophils in humans.^43^ Second, reliance on surrogate endpoints may not guarantee clinical benefit. In the ACCORD Lipid study, adding the PPARα agonist fenofibrate to background statin therapy improved triglyceride and HDL profiles but did not reduce the primary composite cardiovascular outcome in the overall cohort.^767^ Third, clinically significant adverse effects limit the dosing, therapeutic applicability, and patient adherence of some NR modulators, occasionally leading to treatment discontinuation. The FXR agonist OCA frequently causes pruritus and dyslipidemia in patients with MASH.^223^ The LXR agonist BMS-852927 induces lipogenesis and neutropenia, reflecting a class effect that has hindered further clinical development.^43^ Moreover, the RARγ agonist palovarotene causes premature epiphyseal closure in pediatric patients with fibrodysplasia ossificans progressiva (FOP), thereby limiting its clinical applicability.^768^ In addition, patient heterogeneity and geographic variation affect trial outcomes. In the TOPCAT trial of heart failure with preserved ejection fraction, spironolactone did not significantly reduce the primary composite endpoint.^257^ Subsequent analyses revealed that spironolactone showed benefit in the Americas cohort but not in Russia/Georgia, suggesting that geographic and baseline patient differences may dilute overall treatment effects.^769^

By contrast, there are also examples of successful clinical translation. In primary biliary cholangitis, seladelpar (PPARδ) and elafibranor (PPARα/δ) achieved ALP composite response and pruritus improvement in phase 3 studies with a favorable safety profile, underscoring the translational value of disease-specific biochemical and symptom endpoints.^26^^,^^205^ Moreover, OCA has been successfully translated into clinical practice for patients with primary biliary cholangitis, where it received Food and Drug Administration approval in 2016 as a second-line therapy for those with inadequate response or intolerance to ursodeoxycholic acid.^222^^,^^770^ Similarly, in T2D with CKD, finerenone, a nonsteroidal MRA, significantly reduced both renal and cardiovascular composite outcomes on top of background renin-angiotensin system blockade, with hyperkalemia rates generally lower than those with steroidal MRAs.^264^^,^^265^^,^^266^

Collectively, these findings underscore that identifying a “safe-effective” therapeutic window and employing disease-appropriate endpoints are key determinants of successful clinical translation. Accordingly, we summarize several key clinical trials of NRs modulators in Table 3.

**Table 3:** Key clinical trials of NR modulators

NRs Modulators	Indication	Trial/phase/design	Patient population	Primary endpoint(s)	Key outcomes	Selected adverse events
Pioglitazone (PPARγ)	NASH	PIVENS trial: phase 3, multicenter, randomized, placebo-controlled, double-blind, 96 weeks (ClinicalTrials.gov number, NCT00063622)	Non-diabetic adults with biopsy-proven NASH	Histologic improvement, NASH resolution	Improved steatosis and ballooning, improved serum enzyme levels (AST and ALT)	Weight gain^771^
Rosiglitazone (PPARγ)	NASH	FLIRT trial: randomized, double-blind, placebo-controlled, 1 year	Patients with histologically proven NASH	Reduction in steatosis >30% or disappearance of steatosis	Improved steatosis, normalized transaminase levels, improvement in insulin sensitivity, increased serum adiponectin levels	Weight gain, painful swollen legs, reduction in serum hemoglobin level^772^
Seladelpar (PPARβ/δ)	Primary biliary cholangitis	Phase 3, randomized, double-blind, placebo-controlled trial, 12 months (ClinicalTrials.gov number, NCT04620733; EudraCT number, 2020-004348-27)	Patients with PBC who had inadequate response to or intolerance of ursodeoxycholic acid	Biochemical response at month 12: alkaline phosphatase level less than 1.67 times the upper limit of the normal range, with a decrease of 15% or more from baseline, and a normal total bilirubin level	Biochemical response: met primary endpoint criteria, normalization of alkaline phosphatase level, reduction in the score on the pruritus numerical rating scale	COVID-19, headache, abdominal pain, nausea, and abdominal distention^26^
INT131 (PPARγ)	T2D	Randomized, double-blind, placebo- and active-controlled study, 24 weeks (ClinicalTrials.gov number, NCT00631007)	Males or females 30–75 years old with T2D ≥ 6 months on a stable dose (≥3 months) of sulfonylurea with or without metformin, HbA_1c_ 7.5%–10%, and FPG <240 mg/dL	Comparison between treatment groups in change from baseline to week 24 in HbA_1c_	Improvements in glycemic control	Less edema, weight gain, and hemodilution compared with 45 mg pioglitazone^203^
Elafibranor (PPARα/δ)	NASH	International, multicenter, randomized placebo-controlled study, 52 weeks (ClinicalTrials.gov number, NCT01694849)	Patients with NASH without cirrhosis	Reversal of NASH without worsening of fibrosis	NASH resolved without fibrosis worsening, reduced liver enzymes, lipids, glucose profiles, and markers of systemic inflammation	Well tolerated and did not cause weight gain or cardiac events, mild, reversible increase in serum creatinine^204^
Elafibranor (PPARα/δ)	Primary biliary cholangitis	Phase 3, multinational, double-blind, randomized, placebo-controlled trial, ≥52 weeks (ClinicalTrials.gov number, NCT04526665)	Patients with primary biliary cholangitis who had an inadequate response to or unacceptable side effects with ursodeoxycholic acid	Biochemical response (defined as an alkaline phosphatase level of <1.67 times the upper limit of the normal range, with a reduction of ≥15% from baseline, and normal total bilirubin levels)	Biochemical response; normalization of alkaline phosphatase level	Abdominal pain, diarrhea, nausea, and vomiting^205^
Saroglitazar(PPARα/γ)	NAFLD/NASH	EVIDENCES IV study, a multicenter, randomized, double-blind, placebo-controlled phase 2 study, 16 weeks (ClinicalTrials.gov number, NCT03061721)	Patients with NAFLD/NASH with ALT ≥ 50 U/L at baseline and body mass index ≥25 kg/m^2^	The percentage change from baseline in serum ALT levels at week 16	Dose-dependent reduction of ALT level; improved liver fat content and adiponectin, homeostatic model assessment—insulin resistance and triglycerides, lipoprotein particle composition and size; reduced lipotoxic lipid species	Well tolerated^206^
Lanifibranor (pan-PPAR α/δ/γ)	NASH	NATIVE: phase 2b, randomized, double-blind, placebo-controlled trial, 24 weeks (ClinicalTrials.gov number, NCT03008070)	Adults with active NASH	≥2-point decrease in SAF-A score without fibrosis worsening	1200-mg dose met primary endpoint, NASH resolution without fibrosis worsening, fibrosis improvement ≥1 stage without NASH worsening; biomarker (liver enzyme, lipid, inflammatory, and fibrosis) improvements	Diarrhea, nausea, peripheral edema, anemia, weight gain^208^
Obeticholic acid (FXR)	Primary biliary cholangitis	Phase 3, randomized, double-blind, placebo-controlled trial, 12-month (ClinicalTrials.gov number, NCT01473524; Current Controlled Trials number, ISRCTN89514817)	Patients who had an inadequate response to ursodiol or who found the side effects of ursodiol unacceptable to receive OCA at a dose of 10 mg	Alkaline phosphatase level of less than 1.67 times the upper limit of the normal range, with a reduction of at least 15% from baseline, and a normal total bilirubin level	The primary endpoint occurred in achieved in 46% (OCA 5–10 mg) and 47% (OCA 10 mg) vs. 10% (placebo); greater decreases in ALP and total bilirubin; no significant differences in noninvasive fibrosis measures at 12 months	Pruritus, serious adverse events^222^
Obeticholic acid (FXR)	NASH	REGENERATE: phase 3, multicenter, randomized, double-blind, placebo-controlled, 18-month interim (ClinicalTrials.gov number, NCT02548351; EudraCT, 20150-025601-6)	Adults with F2–F3 (and select F1) fibrosis	Fibrosis ≥1-stage improvement without NASH worsening; NASH resolution without fibrosis worsening	Fibrosis improvement; key liver enzymes improvement	Pruritus, increased LDL cholesterol, decreased HDL cholesterol^223^
Tropifexor (FXR)	NASH	Phase 2, randomized, multicenter, double-blind, three-part adaptive design, 48 weeks (ClinicalTrials.gov number, NCT02855164)	Patients with NASH	Safety and tolerability to end of study, and dose response on ALT, AST and HFF at week 12	Decreases from baseline in ALT and HFF	Pruritus^224^
Cilofexor (FXR)	NASH	Phase 2, randomized, double-blind, placebo-controlled, 24 weeks (ClinicalTrials.gov number, NCT02854605)	Adults with NASH	Safety and tolerability; change in steatosis (MRI-PDFF), liver stiffness (MRE, transient elastography), liver biochemistry, noninvasive fibrosis markers, bile acid homeostasis	Reductions in steatosis, bile acids, liver enzymes	Pruritus, abdominal pain, fatigue, nausea, and diarrhea^50^
BMS-852927 (LXR)	Hypercholesterolemia	Phase 1 single (CV201-001) and multiple (CV201-002) ascending dose studies: randomized, double-blind, and placebo-controlled SAD and MAD studies; phase 1b study (CV201008): randomized, site- and subject-blinded, placebo-controlled trial (ClinicalTrials.gov number, NCT01651273)	SAD and MAD studies: Healthy men and women 18–45 years of age with a BMI of 18–30 kg/m^2^; phase 1b study (CV201008): male and female patients between the ages of 18 and 75 years with primary hypercholesterolemia and with a BMI ≤40 kg/m^2^	The effect of multiple doses on LXR target gene induction, plasma lipids, and other safety-related endpoints	Induces blood LXR RCT target genes but elevates plasma and liver lipids and lowers neutrophil counts	Increased plasma and hepatic TG, plasma LDL-C, apoB, apoE, and CETP and decreased circulating neutrophils^43^
Resmetirom (TRβ)	NASH with fibrosis (F2–F3)	MAESTRO-NASH; phase 3, randomized, double-blind, placebo-controlled, 52 weeks (ClinicalTrials.gov number, NCT03900429)	Adults with biopsy-confirmed NASH, F1b–F3	NASH resolution and fibrosis ≥1-stage improvement (co-primary)	Both co-primary endpoints met, ↓ LDL-C; FDA approved 2024	Diarrhea, nausea, statin interactions^181^
Spironolactone (MR)	Patients with kidney failure receiving maintenance dialysis	ACHIEVE, international, parallel-group, randomized controlled trial (ClinicalTrials.gov number, NCT03020303)	Patients were aged 45 years or older, or aged 18 years or older with a history of diabetes and were receiving maintenance dialysis for kidney failure for at least 3 months	Composite of cardiovascular death or hospitalization for heart failure, analyzed as time to the first event	No significant reduction in the primary composite outcome, deaths from any cause and hospitalizations for any cause similar across groups; trial stopped early for futility.	Hyperkalaemia^773^
Spironolactone (MR)	CKD	Prospective, randomized, open, blinded endpoint trial, 3 years of follow-up (ClinicalTrials.gov number, ISRCTN44522369)	Patients with CKD stage 3b	Time from randomization until the first occurrence of death, hospitalization for heart disease, stroke, heart failure, transient ischemic attack or peripheral arterial disease, or first onset of any condition listed not present at baseline	No significant difference in the number of participants who experienced the composite primary outcome	Treatment withdrawn (because of safety concerns: decrease in eGFR, side effects)^256^
Spironolactone (MR)	Heart failure with preserved ejection fraction	Phase 3, multicenter, international, randomized, double-blind, placebo-controlled trial. mean follow-up of 3.3 years (ClinicalTrials.gov number, NCT00094302)	Patients with heart failure and a preserved ejection fraction	Composite of death from cardiovascular causes, aborted cardiac arrest, or hospitalization for the management of heart failure	Spironolactone did not significantly reduce the incidence of the primary composite outcome of death from cardiovascular causes, aborted cardiac arrest, or hospitalization for the management of heart failure	Higher rate of hyperkalemia, increased serum creatinine levels^257^
Eplerenone (MR)	Systolic heart failure	Randomized, double-blind, placebo-controlled trial, median follow-up ∼21 months (ClinicalTrials.gov number, NCT00232180)	Age of at least 55 years, NYHA functional class II symptoms, an ejection fraction of no more than 30% (or, if > 30 to 35%, a QRS duration of >130 ms on electrocardiography), and treatment with an ACE inhibitor, an ARB, or both and a beta-blocker (unless contraindicated) at the recommended dose or maximal tolerated dose	Composite of death from cardiovascular causes or a first hospitalization for heart failure	Primary endpoint occurred in 18.3% in the eplerenone group vs. 25.9% in placebo (HR = 0.63; 95% CI 0.54–0.74; *p* < 0.001). Death from cardiovascular causes and hospitalizations for heart failure and for any cause reduced	Elevated serum potassium (>5.5 mmol/L)^259^
Esaxerenone (CS-3150) (MR)	T2D and microalbuminuria	ESAX-DN study: phase 3, multicenter, randomized, double-blind, placebo-controlled study, 52 weeks (JapicCTI-173695)	Patients with type 2 diabetes and a urinary albumin-to-creatinine ratio of 45 to <300 mg/g creatinine treated with renin-angiotensin system inhibitors	The proportion of patients achieving urinary albumin-to-creatinine ratio remission (<30 mg/g creatinine and ≥30% reduction from baseline on two consecutive occasions)	Achieved urinary albumin-to-creatinine ratio remission, higher percent change in urinary albumin-to-creatinine ratio from baseline to end of treatment, improvement in time to first remission and time to first transition to urinary albumin-to-creatinine ratio ≥300 mg/g creatinine	Serum potassium elevation (≥6.0 or ≥5.5 mEq/L)^263^
Finerenone (MR antagonist)	Patients with CKD and T2D	Phase 3, randomized, double-blind, placebo-controlled, multicenter clinical trial. median follow-up of 2.6 years (ClinicalTrials.gov number, NCT02540993)	Adults (≥18 years of age) with type 2 diabetes and CKD treated with an ACE inhibitor or ARB at the maximum dose on the manufacturer’s label that did not cause unacceptable side effects	Kidney failure, a sustained decrease of at least 40% in the eGFR from baseline, or death from renal causes	Lower incidence of the primary composite outcome of kidney failure, lower risk of a key secondary outcome event (death from cardiovascular causes, nonfatal myocardial infarction, nonfatal stroke, or hospitalization for heart failure)	Higher incidence of hyperkalemia-related discontinuation^266^
Finerenone (MR)	Heart failure with mildly reduced or preserved ejection fraction	International, double-blind, randomized, placebo-controlled trial, median follow-up of 32 months (ClinicalTrials.gov number, NCT04435626)	Patients with heart failure and a left ventricular ejection fraction of 40% or greater	Composite of total worsening heart failure events (with an event defined as a first or recurrent unplanned hospitalization or urgent visit for heart failure) and death from cardiovascular causes	Lower rate of a composite of total worsening heart failure events and death from cardiovascular causes	Increased risk of hyperkalemia and reduced risk of hypokalemia^264^
Palovarotene (RARγ)	Emphysema	REPAIR, investigator-initiated, double-blind, placebo-controlled randomized study, 1 year	Patients with severe α_1_-antitrypsin deficiency and emphysema confirmed by computed tomography	Change in volume-adjusted 15th percentile point lung density from baseline in 1 year	Failed to show a significant benefit on lung density in moderate-to-severe emphysema secondary to severe α_1_-antitrypsin deficiency	Mucocutaneous events^774^
Palovarotene (RAR)	Fibrodysplasia ossificans progressiva	Phase 2, multicenter, randomized, double-blind, sponsor-unblinded, placebo-controlled, dose-ranging trial, 12 weeks (ClinicalTrials.gov number, NCT02190747)	Patients (≥6 years of age) clinically diagnosed with classic FOP; had flare-up onset in an appendicular area, abdomen, or chest within 7 days prior to randomization; and were receiving current standard of care	The proportion of responders in the anterior and lateral projections of the flare-up body region assessed by plain radiograph at week 6	Lower (although not statistically different) volumes of new HO at week 6 and week 12	Dry skin, dry lips, pruritus, erythema, dermatitis acneiform, and dry mouth^768^
Tamoxifen (ER)	Myeloproliferative neoplasms	TAMARIN phase 2, multicenter, single-arm clinical trial (EudraCT 2015-005497-38)	Patients with stable MPNs, no prior thrombotic events and mutated *JAK2*^*V617F*^, *CALR*^*ins5*^, or *CALR*^*del52*^ peripheral blood allele burden ≥20%	≥50% reduction in mutant allele burden at 24 weeks	3 of 38 patients (≈7.9%) achieved the primary endpoint (≥50% allele burden reduction at 24 weeks); secondary: 5/38 achieved ≥25% reduction at 24 weeks; no patient reached ≥50% reduction at 12 weeks	Thrombotic events, toxicity^775^
Tamoxifen (ER)	Breast cancer	Prospective observational multicenter study, 5 years (ClinicalTrials.gov number, NCT00965939)	Postmenopausal women with an estrogen receptor-positive breast cancer	ORR using RECIST criteria 1.0	None of the endpoints was associated with endoxifen levels, tamoxifen metabolites, or TAS.	Hot flashes^776^
Alfacalcidol (VDR)	Hypoparathyroidism	Open-label randomized controlled trial, tertiary care center, 6 months (CTRI/2019/05/019203).	Patients with optimal calcemic control on alfacalcidol were continued on the same (*n* = 20) or switched to calcitriol (*n* = 25) at half of the ongoing alfacalcidol dose	Optimal calcemic control and normalization of hyperphosphatemia and urinary calcium excretion	Comparable serum calcium, phosphate, and calcium excretion; no significant differences in hyperphosphatemia, hypercalciuria, FGF23	Adverse events (serum calcium >10.6 mg/dL, severe hypercalcemia) were infrequent and similar between groups^238^

ACE, angiotensin-converting enzyme; ARB, angiotensin-receptor blocker; AST, aspartate aminotransferase; BMI, body mass index; CETP, cholesteryl ester transfer protein; CI, confidence interval; eGFR, estimated glomerular filtration rate; FOP, fibrodysplasia ossificans progressiva; FPG, fasting plasma glucose; HFF, hepatic fat fraction; HO, heterotopic ossification; HR, hazard ratio; LDL-C, low-density lipoprotein cholesterol; MAD, multiple ascending dose; MRE, magnetic resonance elastography; MRI-PDFF, magnetic resonance imaging-proton density fat fraction; ORR, Objective response rate; PBC, primary biliary cholangitis; SAD, single ascending dose; TG, triglyceride.

### Challenges in NR-targeted therapies and mechanism-linked strategies

NR modulators face recurrent obstacles that span on-target class liabilities, network-level off-target effects, challenging pharmacokinetics, and xenobiotic-receptor-mediated drug-drug interactions.

#### Safety profiles of NR modulators

Balancing efficacy against adverse effects remains challenging across NRs, and many dose-limiting adverse events reflect on-target physiology. FXR agonists show class-consistent pruritus and atherogenic lipid shifts. In MASH trials, OCA was associated with lipid changes and drug-induced liver toxicity consistent with FXR activation^223^^,^^777^ and MET409 explicitly reported on-target HDL-C decreases in humans.^778^ For LXR agonists, systemic activation predictably induces hypertriglyceridemia and steatosis, limiting translation and motivating tissue-selective delivery.^779^ For PPARγ agonists (TZDs), edema, HF, and bone fracture are well-recognized class liabilities that shape indication and patient selection.^780^ For systemic glucocorticoids acting through GR, long-term use increases fractures and infection risk.^781^

#### Off-target liabilities

For systemic GR agonists, clinically important non-target effects arise from ubiquitous GR signaling and dose-time dependence. Contemporary data document bone loss and fragility fractures with oral glucocorticoids and provide up-to-date prevention and treatment guidance.^782^^,^^783^^,^^784^ Large population studies show elevated serious infection risk under systemic glucocorticoids with dose-response features.^785^^,^^786^ Metabolic toxicity is frequent: recent clinical and pharmacoepidemiologic studies describe glucocorticoid-induced hyperglycemia and incident diabetes and related morbidity.^120^^,^^787^ Cardiovascular risk is dose-dependent and detectable even at low dose in immune-mediated diseases.^788^ Additional GC-induced complications include ocular hypertension and glaucoma,^789^ skin atrophy,^790^ and neuropsychiatric adverse effects.^791^ Taken together, these outcomes reflect systemic, pleiotropic GR activation rather than a single-tissue effect.

Beyond direct target pharmacology, NR modulators may engage parallel receptor networks or indirect transcriptional programs. FXR agonists highlight a dual narrative: (1) pruritus is a class and on-target feature^777^^,^^792^ and (2) bile acids can induce itch via TGR5 on sensory neurons and activate TRPA1, suggesting a potential off-target/network amplifier for bile-acid-like or FXR/TGR5-active chemotypes.^793^^,^^794^ Moreover, RXR agonists exhibit intrinsic network pleiotropy because RXR functions as a heterodimeric hub: RXR ligands can amplify signaling through permissive partners such as LXRs, PPARs, and PXR, whereas non-permissive pairings (such as TR/RXR) are not directly driven by RXR ligands.^5^^,^^80^ For example, activation of LXR or RXR upregulates SREBP-1c and downstream lipogenic genes, increasing hepatic triglyceride production and culminating in hypertriglyceridemia.^795^ By contrast, the non-permissive TR/RXR heterodimer underlies a distinct adverse-effect profile: RXR agonists suppress pituitary TSHβ transcription and can cause central hypothyroidism.^796^^,^^797^^,^^798^

#### Pharmacokinetic challenges

Many NR ligands are lipophilic, highly protein bound, and undergo enterohepatic circulation, which complicates exposure-response control. For FXR modulators, two human programs illustrate different ends of the design spectrum. OCA, a semisynthetic bile acid analog, is conjugated to glycine and taurine; OCA and its conjugates undergo enterohepatic recirculation,^799^ a process associated with multiple peaks and a longer apparent half-life in a plasma concentration-time profile.^800^ In contrast, tropifexor (a non-bile-acid FXR agonist) shows no obvious major enterohepatic circulation, exhibits dose-proportional pharmacokinetics, supports once-daily dosing, and demonstrates dose-dependent target engagement.^801^^,^^802^

VDR analogs are typically highly lipophilic and bind vitamin D-binding protein (DBP) with very high affinity, yielding an extremely low free fraction in circulation—approximately 0.03% of 25(OH)D is free; about 85% is DBP bound, and 15% albumin bound—along with prolonged residence in plasma.^803^^,^^804^^,^^805^ The classic manifestation of vitamin D toxicity is hypercalcemia.^806^ These properties underscore the need for careful exposure control and safety monitoring during therapy.

Tamoxifen exemplifies a pharmacokinetic (PK)-pharmacogenomic challenge: formation of the active metabolite endoxifen depends on CYP2D6^807^; inter-patient endoxifen exposure varies widely (up to 20- to 30-fold)^808^ and is linked to clinical outcomes.^807^ Potent CYP2D6 inhibitors substantially reduce endoxifen,^809^ and phenoconversion guidance recommends accounting for inhibitor use when calculating the CYP2D6 activity score.^810^

#### Regulatory considerations of NR modulators and strategies to overcome barriers

The xenobiotic receptors PXR and CAR transcriptionally regulate drug-metabolizing enzymes (such as cytochrome P450) and drug transporters (such as P-glycoprotein), thereby acting as key determinants of drug efficacy, toxicity, and drug-drug interactions.^811^ Accordingly, compounds that directly target PXR/CAR or other NR modulators that secondarily activate PXR/CAR at certain exposures or with specific chemotypes may induce or inhibit these enzymes and transporters, altering the exposure, effect, and toxicity of co-medications.

PK architecture itself can challenge exposure-response control. Enterohepatic circulation produces multi-peak concentration-time profiles and distorts conventional exposure metrics (such as AUC), underscoring the value of model-informed analyses when planning dose regimens and interpreting safety signals.^800^^,^^812^ In parallel, many NRs ligands are small molecule, hydrophobic, and highly protein bound; together with lipophilicity-driven tissue partitioning and population differences (such as body fat composition, protein binding levels, disease conditions), these properties magnify exposure variability and motivate quantitative modeling for drug-drug interactions and special populations.^813^^,^^814^^,^^815^ Consistent with recent practice, integrating physiologically based pharmacokinetic (PBPK) approaches within model-informed drug development (MIDD) supports dose optimization and drug-drug interactions assessment.^816^^,^^817^

A recurring on-target liability for specific NR classes also drives regulatory scrutiny. For instance, systemically active LXR agonists activate SREBP-1c-linked lipogenesis, driving hepatic triglyceride production, steatosis, and hypertriglyceridemia. A practical mitigation is tissue-selective delivery to re-distribute exposure: for example, synthetic HDL nanoparticles that deliver LXR agonists to plaque macrophages preserved atheroprotective actions, while limiting hepatic lipid effects.^779^ Related nanomedicine studies similarly describe macrophage-targeted LXR formulations that reduce plaque burden while attenuating hepatic lipogenesis-related side effects,^818^ supporting exposure re-distribution as a class-specific mitigation strategy.

For FXR, systemic adverse effects observed in trials have motivated non-bile-acid chemotypes and intestinally biased modulators (such as tropifexor and cilofexor) to improve tolerability, while maintaining pharmacodynamics, which achieve tissue selectivity through medicinal-chemistry strategies.^819^

Biased agonism and non-classical NR modulation offer a route to decouple efficacy from dose-limiting toxicities. For PPARγ, distinct agonists can bias coactivator recruitment and downstream signaling, suggesting designs that retain efficacy with fewer off-pathway liabilities.^820^ These approaches collectively align with the regulatory aims of predictable exposure, minimized interaction risk, and mechanism-linked mitigation, without sacrificing the therapeutic promise inherent to NR biology.

### Conclusion and perspectives

NRs act as key integrators of endocrine, nutritional, and inflammatory signals to regulate metabolic and immune homeostasis and control the pathogenesis of related diseases. Notably, individual NRs exert context-specific effects—for example, the dual metabolic and anti-inflammatory roles of PPARs, the lipogenic vs. anti-atherogenic actions of LXRs, and the cell-specific immunomodulatory functions of GR—highlighting both the complexity and therapeutic potential of NR-based interventions.

Several synthetic or natural NRs ligands—including selective modulators, agonists, inverse agonists, and antagonists—have demonstrated therapeutic efficacy across preclinical models and clinical settings. Agents such as PPARs agonists, FXR modulators, TRβ-selective compounds, MRAs, and SERMs are already used or under active development for conditions ranging from metabolic syndrome and immune-related disease. However, clinical application of NR-targeting therapies faces challenges related to off-target effects, limited tissue selectivity, and adverse metabolic consequences (such as steatosis, hyperlipidemia, or immunosuppression).

Looking forward, next-generation NR therapeutics should pair exposure redistribution via tissue-selective delivery, chemotype redesign with organ-biased pharmacology, and biased agonism/non-classical modulation to decouple efficacy from toxicity. These discovery strategies ought to be integrated with model-informed development to manage drug-drug interactions, special populations, and exposure-response heterogeneity, and combined with systems profiling (multi-omics, single-cell) and structural biology to map NRs crosstalk, guide patient stratification, and refine biomarkers. Together, these principles align with regulatory priorities—predictable exposure, minimized interaction risk, and mechanism-linked risk mitigation—and position NR pharmacology for precision medicine in metabolic and cardiovascular diseases.

## Acknowledgments

This study was supported by grant from the 10.13039/501100012166National Key Research and Development Program of China (2022YFA1105104), the 10.13039/501100001809National Natural Science Foundation of China (82070358, 82470433, and 81770430) and Shanghai Committee of Science and Technology (24ZR1446100).

## Author contributions

F.L., Y.D., and L.L. contributed to conceptualization. F.L. and Q.C. wrote the original draft. Q.C. contributed to visualization. Y.D. and L.L. supervised the work and were responsible for writing – review and editing. All authors reviewed and approved the final manuscript.

## Declaration of interests

The authors declare no competing interests.
